# From molecular design to antiepileptic evaluation: sulfonamide–pyrazole derivatives as promising neuroactive agents

**DOI:** 10.1039/d6ra01165a

**Published:** 2026-05-26

**Authors:** Mohamed K. Elgohary, Mahmoud Abdelrahman Alkabbani, Aya Mohamed Ahmed Ibrahim, Ahmed Elsonbaty, Abdelhameed Abubakr, Mayada H. Mohamed, Abdulrahman A. Almehizia, Ahmed M. Naglah, Mohamed Fares, Hatem A. Abdel-Aziz

**Affiliations:** a Pharmaceutical Chemistry Department, Faculty of Pharmacy, Egyptian Russian University Badr City Cairo 11829 Egypt mohamed-elgohary@eru.edu.eg; b Pharmacology and Toxicology Department, Faculty of Pharmacy, Egyptian-Russian University Badr City Cairo 11829 Egypt; c Department of Biochemistry, Faculty of Pharmacy, Egyptian Russian University Badr City Cairo Egypt; d Department of Pharmaceutics and Pharmaceutical Technology, Egyptian Russian University Badr City Cairo Egypt; e University Family Medicine Center, Department of Family and Community Medicine, College of Medicine, King Saud University Medical City P. O. Box 2925 Riyadh 11472 Saudi Arabia; f Drug Exploration and Development Chair (DEDC), Department of Pharmaceutical Chemistry, College of Pharmacy, King Saud University P. O. Box 2457 Riyadh 11451 Saudi Arabia anaglah@ksu.edu.sa; g School of Pharmacy, The University of Sydney Sydney NSW 2006 Australia; h Applied Organic Chemistry Department, National Research Center Dokki Cairo 12622 Egypt

## Abstract

Neuroinflammation, oxidative stress, and glutamate-mediated excitotoxicity are central pathological mechanisms underlying epileptogenesis and seizure propagation. In the present study, a series of rationally designed sulfonamide pyrazole derivatives was evaluated for their anticonvulsant potential using pentylenetetrazol (PTZ)- and pilocarpine-induced seizure models in mice. Among the tested compounds, compound 6d emerged as the most promising candidate, exhibiting superior anticonvulsant efficacy. In the PTZ model, compound 6d afforded 90% seizure protection with complete survival, outperforming the reference drug sodium valproate. In the pilocarpine-induced status epilepticus model, compound 6d significantly prolonged seizure onset latency, markedly suppressed seizure severity (reducing Racine scores by >80%), and ensured 100% survival. Mechanistic investigations revealed that compound 6d exerted pronounced neuroprotective effects in hippocampal tissue by significantly attenuating oxidative stress (malondialdehyde and nitrite levels), neuroinflammation (TNF-α and IL-6), and excitotoxicity (glutamate levels), with greater efficacy than valproate. Importantly, sub-chronic oral administration of compound 6d did not induce detectable hepatic, renal, or cardiac toxicity, indicating a favorable preliminary safety profile. Collectively, these findings identify compound 6d as a promising lead anticonvulsant agent with multimodal neuroprotective actions and support its further preclinical development as a potential disease-modifying therapy for epilepsy.

## Abstract 2

Neuroinflammation, oxidative stress, and glutamate-mediated excitotoxicity are central pathological mechanisms underlying epileptogenesis and seizure propagation.

## Graphical abstract

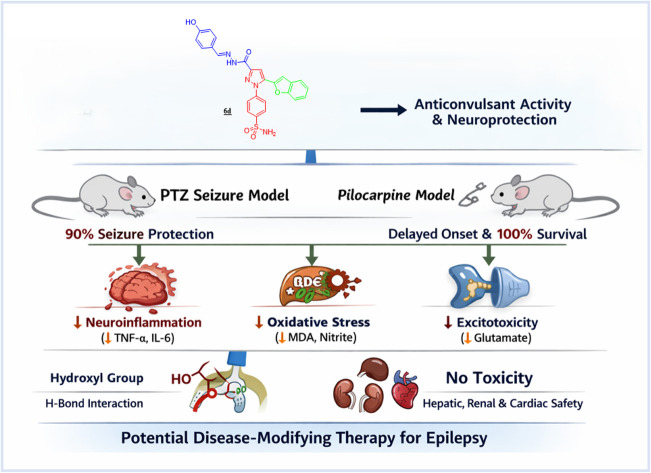

## 1 Introduction

Epilepsy is a prevalent chronic neurological disorder affecting over 50 million people worldwide, representing nearly 1% of the global population.^1^ It is second only to stroke in prevalence among neurological conditions and ranks as one of the most significant contributors to the worldwide burden of brain disorders. Beyond its hallmark seizures, epilepsy carries profound health, social, and economic consequences, placing a heavy strain on patients, caregivers, and healthcare systems.^2^ Although epilepsy has been recognized for centuries, significant progress in its understanding, diagnosis, and treatment has only occurred in recent decades. Despite the availability of numerous antiepileptic drugs (AEDs), approximately one-third of patients remain refractory to treatment, continuing to experience uncontrolled seizures and highlighting the urgent need for more effective therapeutic strategies.

**Fig. 1:**
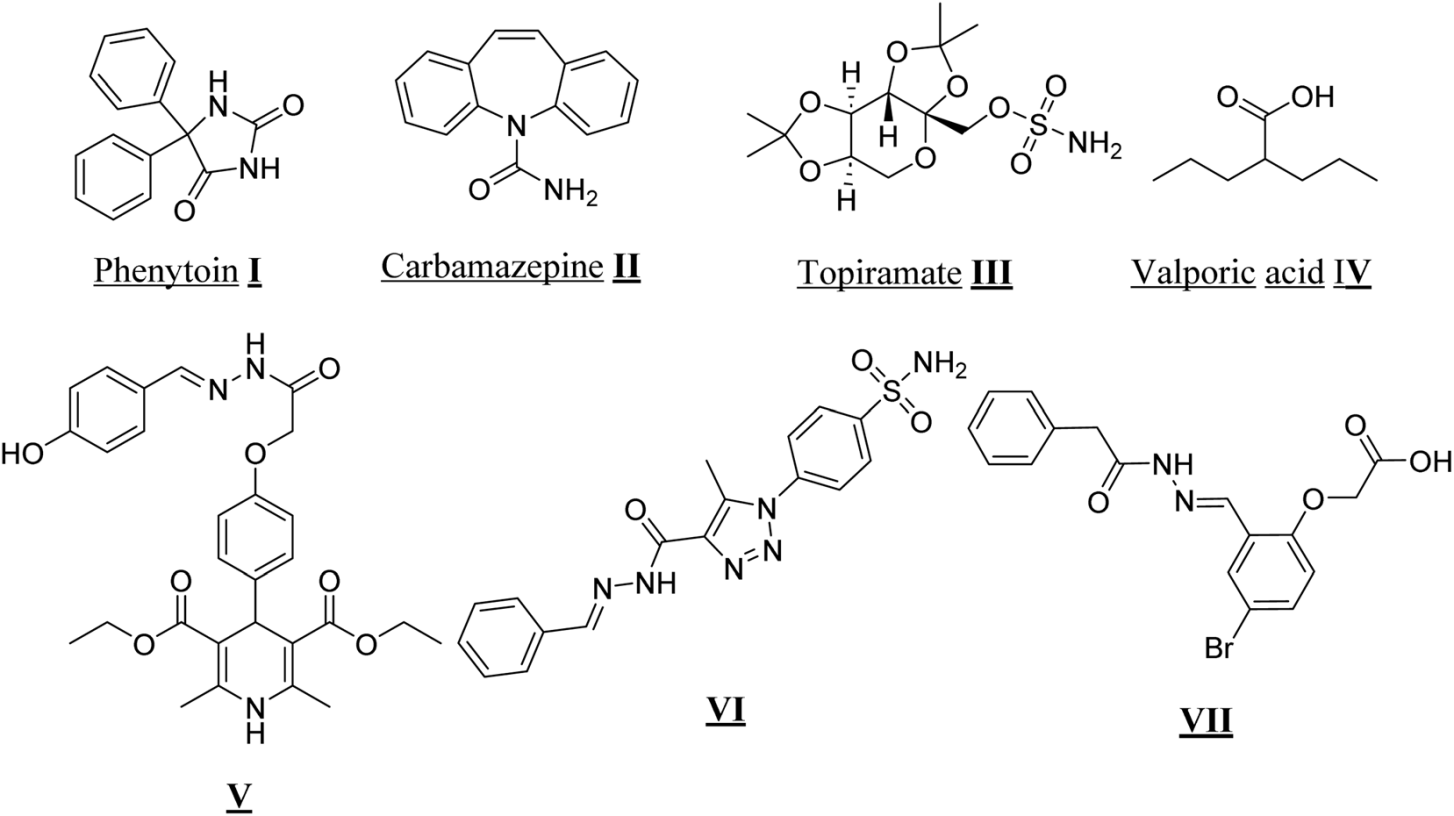
Diagram of marketed compounds I–IV, and previously reported compounds V–VII, as antiepileptic candidates.

The management of epilepsy predominantly relies on long-term pharmacotherapy, with surgical interventions reserved for select patients in whom medications fail to provide adequate seizure control.^3^ Conventional antiepileptic drugs (AEDs), including phenytoin (I) and carbamazepine (II), act *via* diverse mechanisms to suppress neuronal hyperexcitability and prevent seizure recurrence. Despite their widespread clinical use, these agents often demonstrate limited efficacy in drug-resistant populations and are associated with undesirable adverse effects, underscoring the need for safer and more effective therapeutic options,^4,5^ (Fig. 1).

Sulfamate-substituted drugs, such as topiramate (III), have shown efficacy across multiple seizure types, representing an important advancement in clinical management. More recently developed AEDs, including valproic acid (IV), levetiracetam, and lamotrigine, have further expanded the therapeutic landscape, however, many of these agents still produce adverse effects such as hepatotoxicity, hirsutism, nausea, and weight gain.^6^ Furthermore, drug resistance and neurotoxicity remain significant barriers to optimal seizure control,^7^ highlighting the persistent need for novel anticonvulsant agents with enhanced efficacy and improved safety profiles (Fig. 1).

In contemporary medicinal chemistry, efforts are increasingly directed toward the rational design of compounds with greater target selectivity and optimized pharmacological properties. Mechanistically, AEDs primarily exert their effects by potentiation of GABAergic inhibitory transmission, suppression of glutamatergic excitatory signaling, or modulation of voltage-gated sodium and T-type calcium channels.^8^ Such targeted approaches are essential for developing next-generation antiepileptic agents capable of overcoming the limitations of existing therapies.

Recent literature suggests that the mechanisms of action of many currently available antiepileptic drugs (AEDs) remain incompletely understood. While some AEDs do not exhibit specific receptor-binding interactions, others act through diverse and sometimes poorly defined pathways.^9,10^ This lack of selectivity and clarity in mechanism can contribute to suboptimal efficacy and undesirable side effects, underscoring the urgent need for the development of novel therapeutic agents with precise molecular targets and improved safety profiles. Consequently, contemporary medicinal chemistry research has increasingly focused on designing new anticonvulsant compounds that not only provide better seizure control but also overcome the limitations associated with existing therapies.^11^

Inflammation, the body's natural defense response to harmful stimuli such as trauma, infection, or chemical irritants, has been recognized as a key contributor to the pathophysiology of epilepsy.^12^ Clinically, inflammatory processes manifest as heat, swelling, redness, pain, and impaired tissue function.^13^ At the molecular level, inflammation involves the activation and migration of leukocytes, increased vascular permeability, and the release of various pro-inflammatory mediators. Critical enzymes, including cyclooxygenases (COX) and lipoxygenases, drive the production of prostaglandins, leukotrienes, and reactive oxygen species (ROS), which collectively propagate and amplify the inflammatory cascade.^14^ Understanding these pathways highlights the potential for designing multifunctional compounds that combine anticonvulsant and anti-inflammatory activities, offering a promising strategy for more effective epilepsy treatment.

Hybrid molecules, which integrate multiple pharmacophoric units into a single chemical scaffold, have attracted considerable interest in drug discovery due to their potential for enhanced biological activity and multi-target interactions. Within this framework, hydrazone derivatives have emerged as promising candidates, particularly when combined with benzene sulfonamide and pyrazole moieties through molecular hybridization and bioisosteric modifications. This approach not only enhances synthetic accessibility but also broadens the pharmacological profile of the resulting compounds.^15^

Importantly, several hydrazone-based derivatives containing structurally distinct moieties V–VII have demonstrated significant anticonvulsant activity through diverse mechanisms of action, highlighting their relevance in the ongoing search for novel antiepileptic agents.^16–18^

By strategically incorporating benzenesulfonamide cores with flexible hydrogen-bonding or cyclic hydrazide linkers, along with pyrazole and additional aromatic substituents, these hybrid molecules are designed to optimize target interactions, enhance binding affinity, and provide multifunctional activity, making them promising candidates for next-generation anticonvulsant therapy (Fig. 1).

## 2 Rational and design

**Fig. 2:**
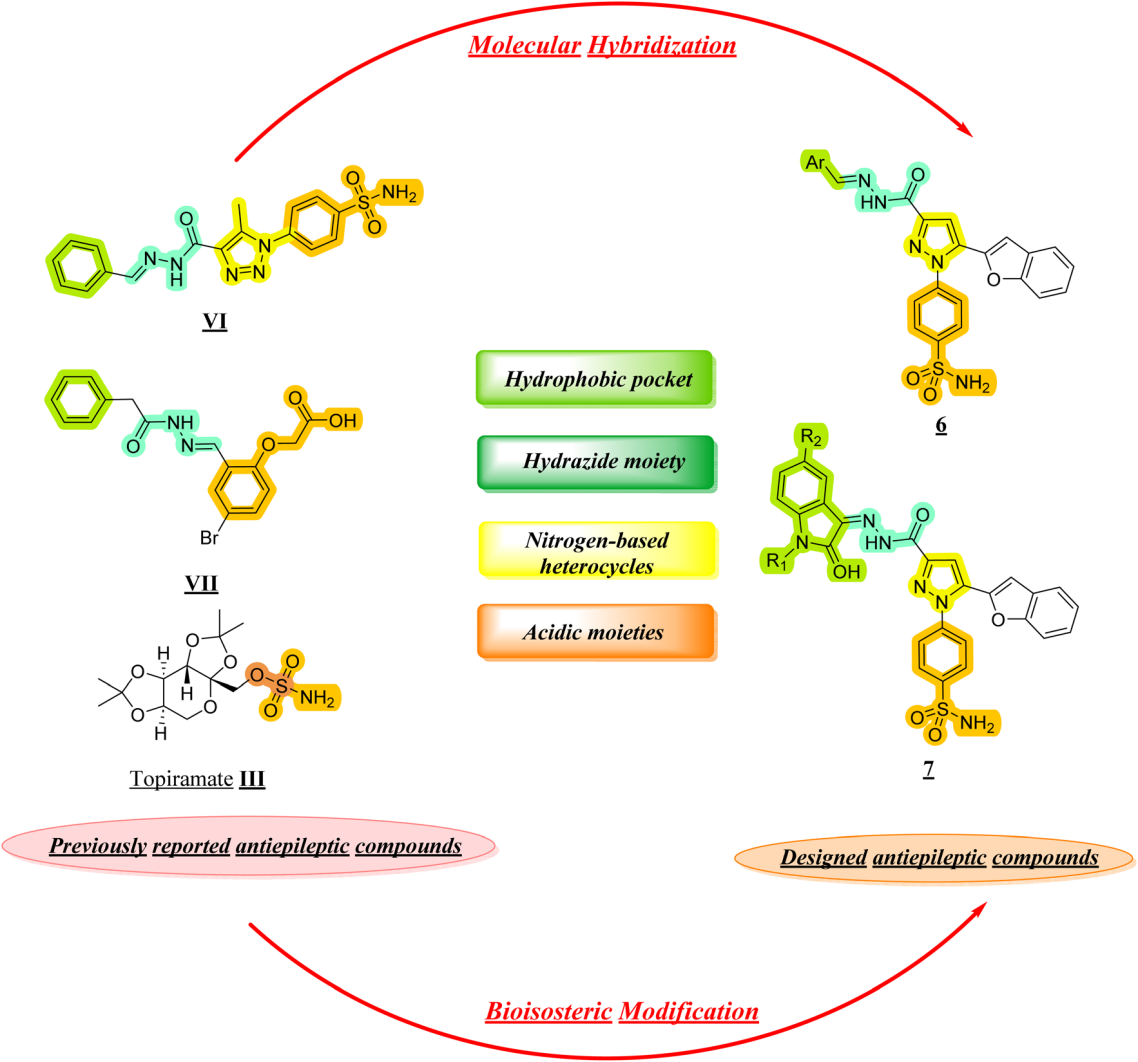
Rational design strategy of dual antiepileptic and anti-inflammatory agents 6b–d, 7a, 7e, and 7f.

Building upon our previously published work on anti-inflammatory scaffolds, the current study extends this framework through a rational repurposing strategy aimed at exploring their antiepileptic potential. As illustrated in the referenced Fig. 2, the design concept was derived from earlier reported benzenesulfonamide-based derivatives, which were strategically modified *via* molecular hybridization and bioisosteric replacement to enhance their neuroactive profile. Specifically, the previously reported anti-inflammatory pharmacophores were integrated with hydrogen-bonding linkers or cyclic hydrazide moieties, alongside the incorporation of a pyrazole ring and additional aromatic substitutions to improve hydrophobic and binding interactions within the target site. Furthermore, bioisosteric transformation, including the replacement of the triazole ring with a pyrazole scaffold and the introduction of isatin-based moieties, was employed to optimize biological activity. This rational evolution from our prior study highlights the structural continuity while emphasizing the novelty of the current design, ultimately leading to the identification of promising candidates 6b–d, 7a, 7e, and 7f with dual anti-inflammatory and anticonvulsant potential, as supported by *in vivo* and biochemical evaluations.

## 3 Bioinformatics study

### 3.1 Identification of overlapping targets and hub genes between active compound and anti-epilepsy-related targets

**Fig. 3:**
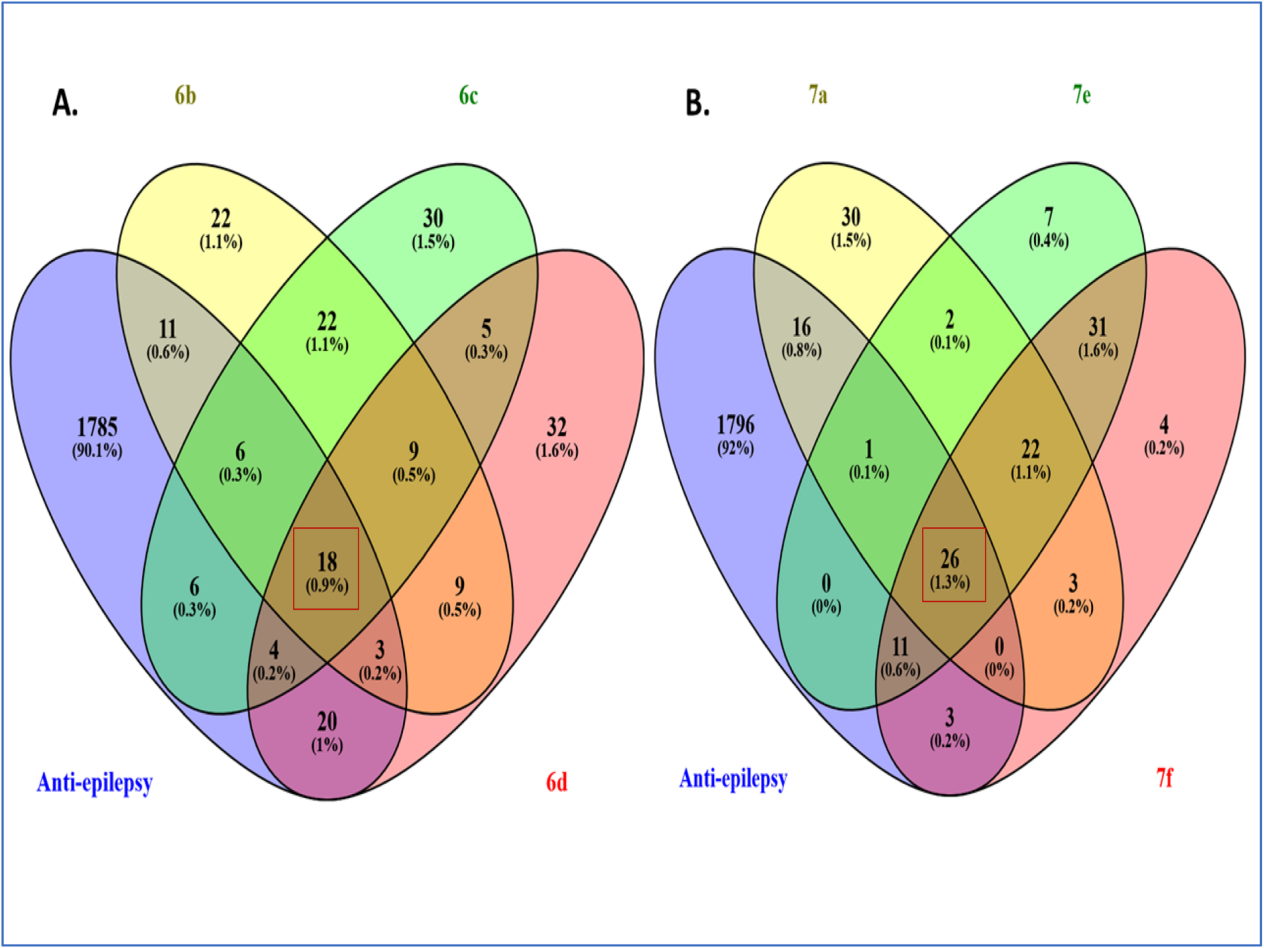
Overlap between compounds 6b–d, 7e, and 7f, with anti-epilepsy–associated targets. (A) Venn diagram illustrating the overlapping targets between compounds 6b–6d and anti-epilepsy-associated target genes. (B) Common targets shared between compounds 7a, 7e, 7f and anti-epilepsy-associated targets.

A total of 1853 anti-epilepsy-associated targets were retrieved from the GeneCards and DisGeNET databases. These targets were intersected with the top 100 predicted targets for each group of compounds 6b–d, 7a and 7e, 7f, resulting in 18 and 26 overlapping genes, respectively, as illustrated in the Venn diagrams (Fig. 3A and B).

### 3.2 Integrated anti-epileptic network analysis of predicted compound targets

The KEGG pathway enrichment analysis was performed to explore anti-epilepsy-related signaling pathways potentially targeted by the investigated compounds. As shown in Table 1, compounds 6b, 6c, and 6d were significantly enriched in pathways related to epidermal growth factor receptor (EGFR) tyrosine kinase inhibitor resistance, vascular endothelial growth factor (VEGF) signaling pathway, erythroblastic oncogene B (ErbB) signaling pathway, and Ras-associated protein-1 (Rap1) signaling pathway. These pathways exhibited high fold enrichment values and statistically significant false discovery rate (FDRs), indicating a strong involvement of receptor-mediated and intracellular signaling mechanisms.

**Table 1:** Functional enrichment analysis of predicted targets associated with the antiepileptic potential of the investigated compounds (6b, 6c, 6d)^a^

Enrichment FDR	*n*Genes	Pathway genes	Fold enrichment	Pathways
9.1 × 10^−14^	8	79	130.8	EGFR tyrosine kinase inhibitor resistance
9.2 × 10^−9^	5	59	109.5	VEGF signaling pathway
1.7 × 10^−11^	7	84	107.7	ErbB signaling pathway
2.1 × 10^−9^	7	211	42.9	Rap1 signaling pathway

a Pathway genes represent the total number of genes annotated in the corresponding KEGG pathway. Fold enrichment reflects the degree of overrepresentation of target genes within each pathway. FDR denotes the false discovery rate. FDR, false discovery rate; *n*Genes, number of enriched genes, EGFR, epidermal growth factor receptor; ErbB, erythroblastic oncogene B; VEGF, vascular endothelial growth factor; HIF-1, hypoxia-inducible factor-1; Rap1, Ras-associated protein-1.

In Table 2, compounds 7a, 7e, and 7f demonstrated enrichment in multiple kinase-associated pathways, including EGFR tyrosine kinase inhibitor resistance, ErbB signaling pathway, VEGF signaling pathway, hypoxia-inducible factor-1 (HIF-1) signaling pathway, Rap1 signaling pathway, phosphoinositide 3-kinase/protein kinase B (PI3K-Akt) signaling pathway, and mitogen-activated protein kinase (MAPK) signaling pathway. Notably, these pathways showed higher gene counts and broader pathway coverage compared with compounds 6b–6d.

**Table 2:** The KEGG enrichment of anti-epileptic–related target genes for compounds (7a, 7e, 7f)^a^

Enrichment FDR	*n*Genes	Pathway genes	Fold enrichment	Pathways
7.0 × 10^−15^	9	79	101.9	EGFR tyrosine kinase inhibitor resistance
9.0 × 10^−15^	9	84	95.8	ErbB signaling pathway
6.8 × 10^−10^	6	59	91	VEGF signaling pathway
4.5 × 10^−10^	7	109	57.4	HIF-1 signaling pathway
4.3 × 10^−13^	10	211	42.4	Rap1 signaling pathway
1.8 × 10^−12^	11	362	27.2	PI3K-Akt signaling pathway
8.0 × 10^−9^	8	300	23.9	MAPK signaling pathway

a Pathway genes represent the total number of genes annotated in the corresponding KEGG pathway. Fold enrichment reflects the degree of overrepresentation of target genes within each pathway. FDR denotes the false discovery rate. FDR, false discovery rate; *n*Genes, number of enriched genes, EGFR, epidermal growth factor receptor; ErbB, erythroblastic oncogene B; VEGF, vascular endothelial growth factor; HIF-1, hypoxia-inducible factor-1; Rap1, Ras-associated protein-1; PI3K-Akt, phosphoinositide 3-kinase/protein kinase B; MAPK, mitogen-activated protein kinase; EGFR-TKI, epidermal growth factor receptor tyrosine kinase inhibitor.

**Fig. 4:**
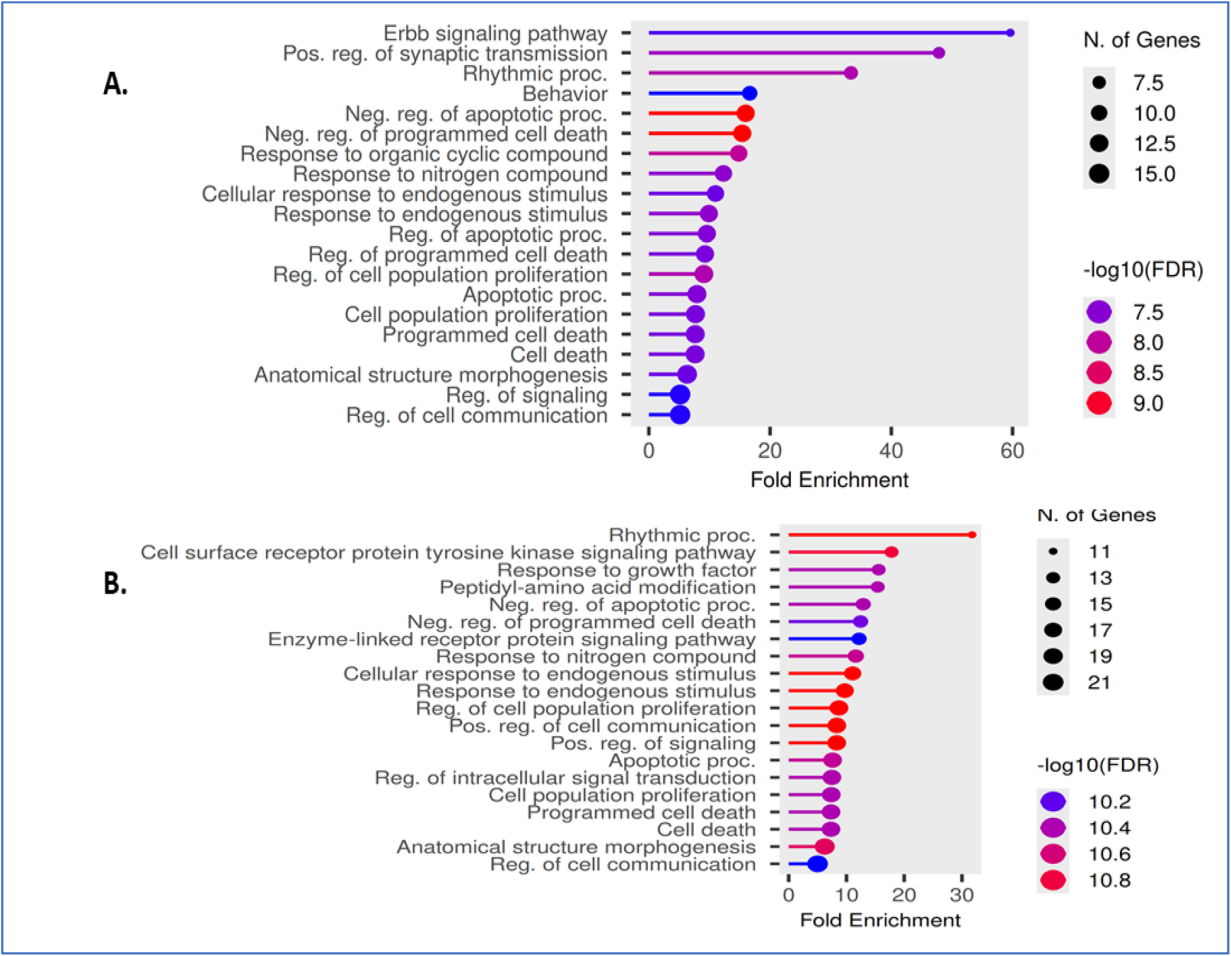
Gene ontology (GO) biological process enrichment analysis of compounds evaluated for potential antiepileptic activity. (A) Compounds 6b, 6c, and 6d, showing enrichment in biological processes related to regulation of apoptosis, programmed cell death, and cellular signaling. (B) Compounds 7a, 7e, and 7f, enriched in receptor-mediated signaling pathways, rhythmic processes, and regulation of cell communication. Nodes represent enriched pathways; node size indicates enrichment strength. Edges show shared genes/functional overlap. Clusters highlight coordinated immune and ECM-related signaling. Dot size represents the number of genes, color indicates −log10(FDR), and the *x*-axis denotes fold enrichment. Analysis was performed using Shiny GO.

As shown in Fig. 4A, gene ontology (GO) biological process enrichment analysis of compounds were predicted to influence processes including the regulation of apoptosis, programmed cell death, cell communication, receptor-mediated signaling, and rhythmic processes. Such these processes are directly relevant to neuroprotection and seizure modulation, implying that these compounds could counteract neuronal hyperexcitability associated with epilepsy.

In Fig. 4B, compounds 7a, 7e, and 7f exhibited prominent enrichment in biological processes associated with cell surface receptor protein tyrosine kinase signaling, enzyme-linked receptor protein signaling pathway, and rhythmic process. Moreover, enrichment was observed in processes related to regulation of cell communication, apoptotic signaling, and cell death, suggesting modulation of receptor-mediated signaling and neuronal synchronization.

**Fig. 5:**
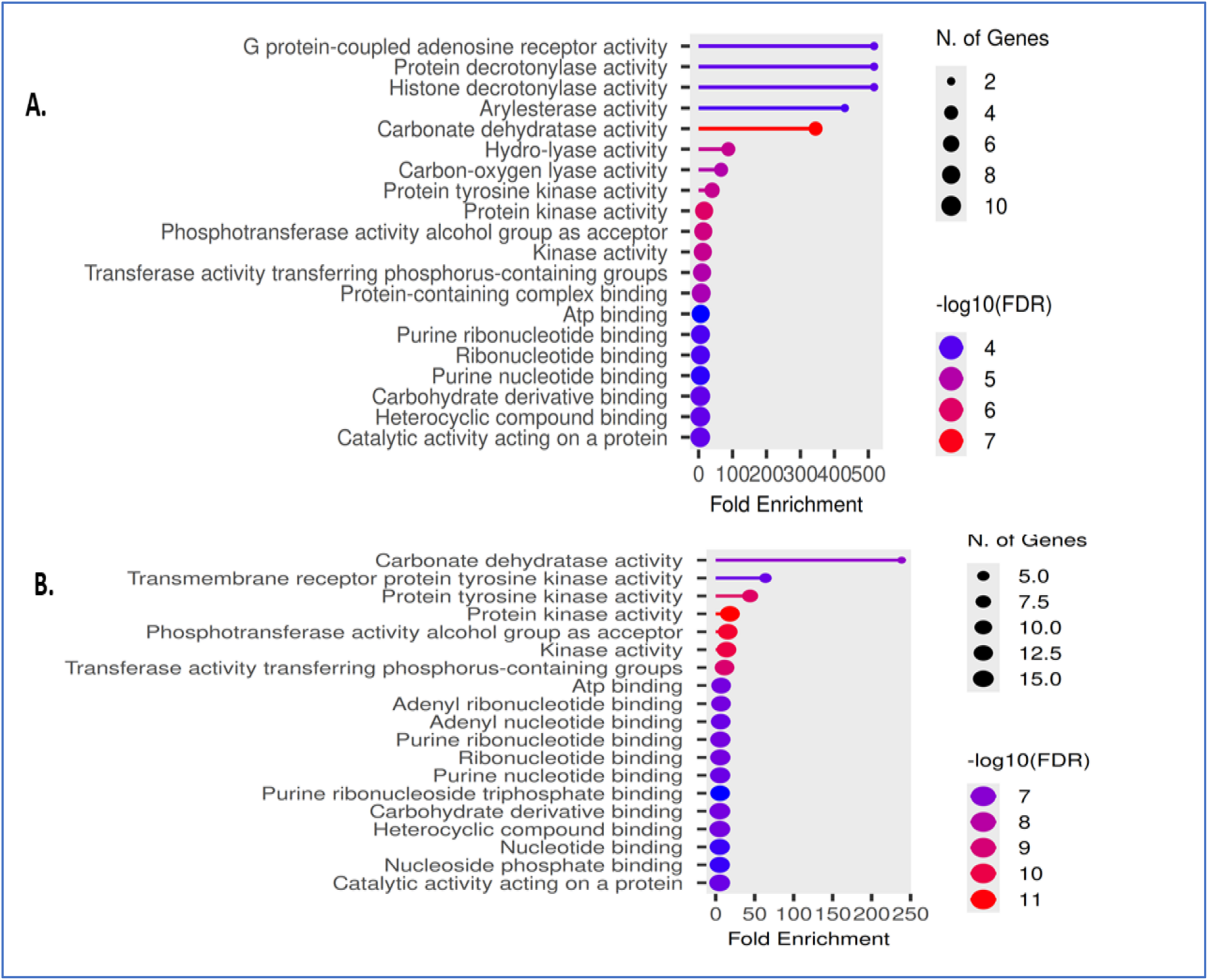
Gene ontology (GO) molecular function enrichment analysis of compounds evaluated for potential antiepileptic activity. (A) GO molecular function network for compounds 6b, 6c, 6d. (B) GO molecular function network for compounds 7a, 7e, 7f. Nodes represent enriched pathways; node size indicates enrichment strength. Edges show shared genes/functional overlap. Dot size represents the number of genes, color indicates −log10(FDR), and the *x*-axis shows fold enrichment. Analysis was performed using Shiny GO.

To evaluate the potential antiepileptic relevance of the investigated compounds, gene ontology (GO) molecular function enrichment analysis was conducted using Shiny GO. Compounds were grouped as 6b, 6c, and 6d (Fig. 5A). These compounds demonstrated significant enrichment in molecular functions associated with G protein-coupled adenosine receptor activity, histone deacetylase activity, and phosphotransferase activity transferring phosphorus-containing groups. Additional enrichment was observed in protein kinase activity and ATP binding, indicating involvement in signaling and regulatory processes relevant to neuronal excitability and 7a, 7e, and 7f (Fig. 5B). Compounds were predominantly enriched in transmembrane receptor protein kinase activity, protein tyrosine kinase activity, and phosphotransferase activity, along with purine ribonucleotide binding and other nucleotide-related functions. These pathways showed comparatively higher fold enrichment values, suggesting strong engagement of phosphorylation-dependent signaling mechanisms.

## 4 Predicted toxicological profile

**Fig. 6:**
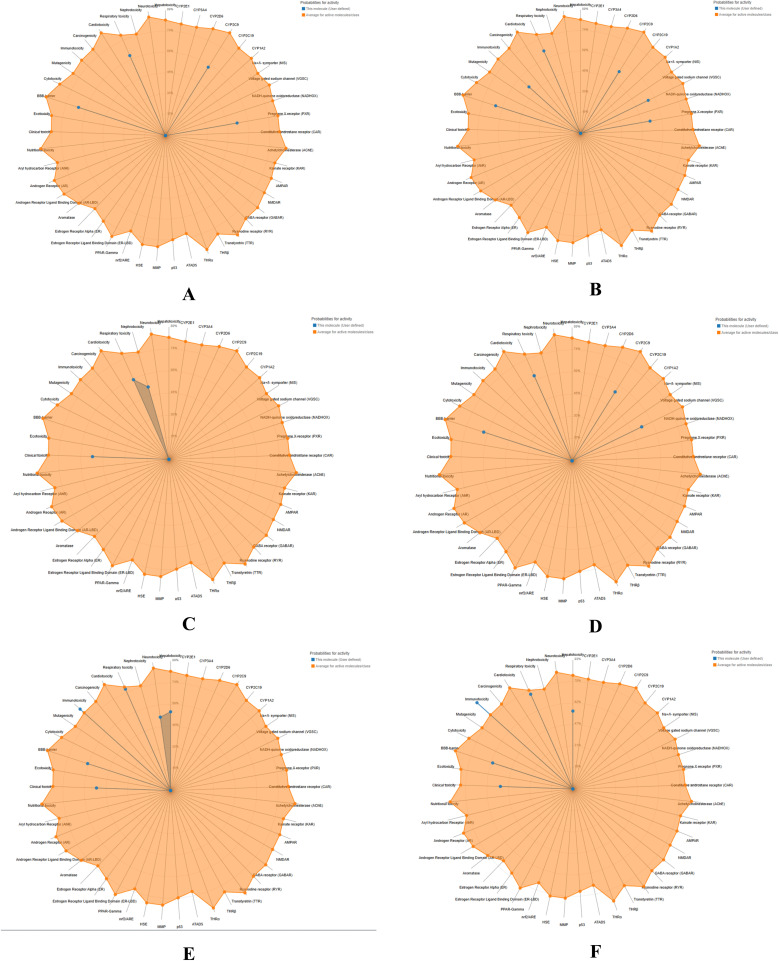
Toxicity radar of the designed compounds predicted using the ProTox-3.0 software 6b (A), 6c (B), 6d (C), 7a (D), 7e (E), and 7f (F).

ProTox-3.0 *in silico* predictions indicated that compounds 6b–d, 7a, 7e, and 7f belong to toxicity class VI, with estimated LD_50_ values ranging from approximately 500 to 3000 mg kg^−1^. These results suggest a low risk of acute oral toxicity and a wide therapeutic safety margin. Consistently, toxicity radar chart profiling and active toxicity cluster analyses confirmed the overall low toxicological risk of the designed compounds (Fig. 6 and S1).

## 5 Pathophysiology of neuroinflammation

**Fig. 7:**
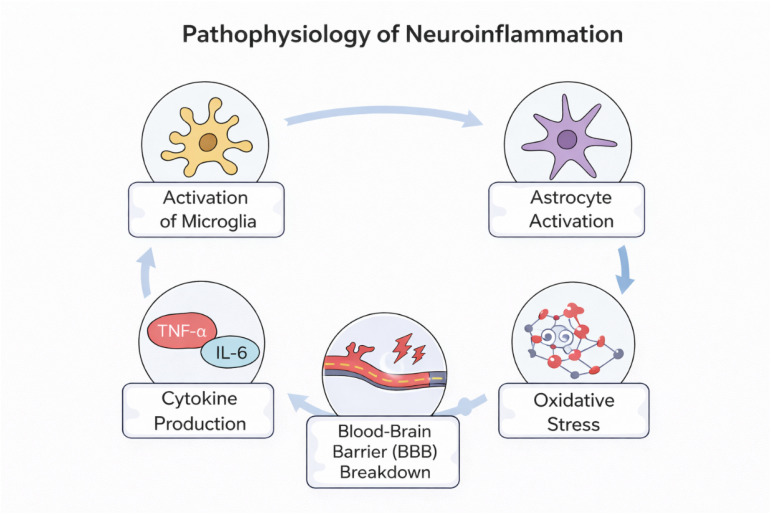
Neuroinflammatory and oxidative stress cascade in epilepsy.

Seizure-induced neuronal hyperexcitability disrupts the integrity of the blood–brain barrier (BBB), leading to increased permeability.^19^ This process is initiated primarily by glutamate-mediated activation of NMDA receptors. BBB dysfunction is accompanied by altered expression of ATP-binding cassette transporters, particularly P-glycoprotein (P-gp), which is markedly upregulated following seizure activity both at the BBB and within brain tissue. P-gp actively effluxes antiepileptic drugs from the brain, thereby reducing their effective intracerebral concentrations.^20^ Mechanistic studies reveal that glutamate-induced P-gp overexpression is mediated through NMDA receptor activation and downstream cyclooxygenase-2 (COX-2) signaling, and that pharmacological inhibition of COX-2 or blockade of EP1 receptors can attenuate seizure-induced P-gp upregulation.^21^ These findings collectively highlight COX-2 as a key regulator of BBB dysfunction and a critical contributor to pharmacoresistance in epilepsy (Fig. 7).

## 6 Results and discussion

### 6.1 Chemistry

The design and synthesis of the target compounds 6b–d, 7a, 7e, and 7f were carried out following the synthetic pathways and experimental protocols described in our earlier study^22^ (SI).
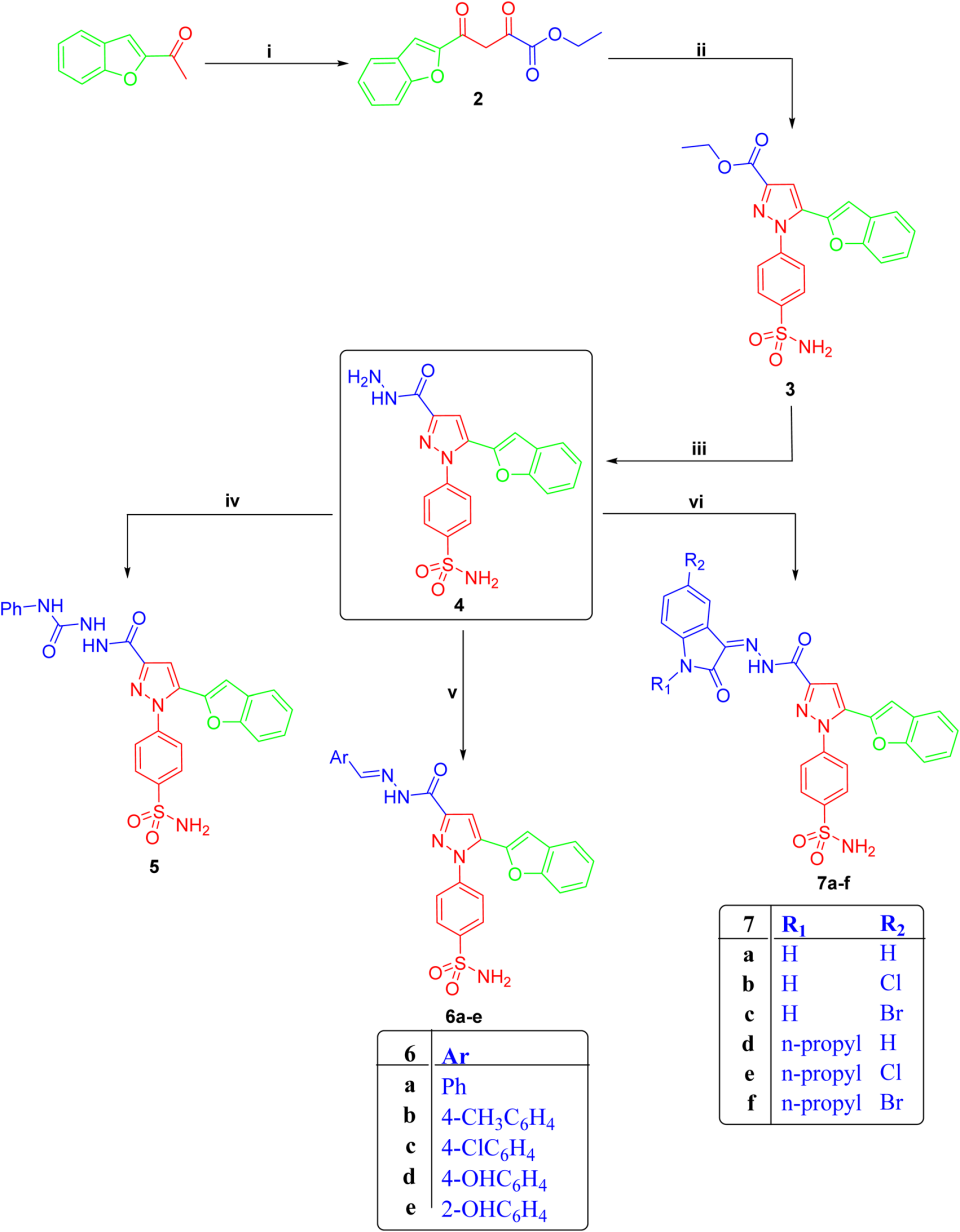


Scheme: reagents and conditions: (i) (COOEt)_2_/NaOC_2_H_5_/EtOH/0 °C; (ii) *p*-SO_2_NH_2_C_6_H_4_NHNH_2_/EtOH, AcOH/reflux 3 h; (iii) N_2_H_4_·H_2_O/reflux 3 h; (iv) phenyl isothiocyanate/EtOH reflux 1 h, (v) EtOH/few drops AcOH/reflux 4 h, (vi) EtOH, few drops AcOH/reflux 4 h.

## 7 Biological study

Guided by the mechanistic insights into the self-perpetuating pathological cycle of neuroinflammation, oxidative stress, and neuronal hyperexcitability in epilepsy, our design strategy prioritized the evaluation of the most potent candidates 6b–d, 7a, 7e, and 7f, which demonstrated pronounced COX-2 inhibitory activity. These selected compounds were subsequently investigated for their anticonvulsant efficacy by targeting key molecular and cellular pathways involved in seizure initiation and propagation. The underlying rationale is that pharmacological inhibition of COX-2 may attenuate the neuroinflammatory cascade by suppressing pro-inflammatory cytokine release, mitigating oxidative stress, and stabilizing neuronal excitability, thereby conferring dual therapeutic benefits as an anti-inflammatory and an antiepileptic agent.

## 8 Anti-inflammatory assay

### 8.1 *In vitro* COX-2 assay^22^

In line with our previously published findings, compounds 6b–d, 7a, 7e, and 7f showed potent and selective inhibitory activity against the COX-2 isozyme, with IC50 values of 0.07–0.09 µM, comparable to or exceeding those of celecoxib (Table 3).

**Table 3:** *In vitro* inhibitory activity of designed compounds 6b–d, 7a, 7e, and 7f toward COX-2 enzymes

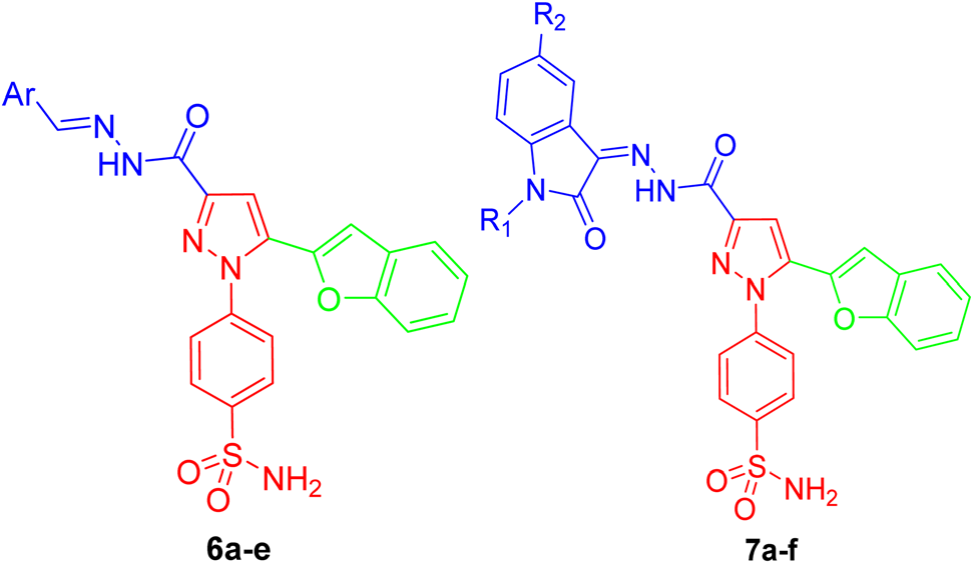					
Compound	X	R_1_	R_2_	COX-2 IC_50_ (µM)	SI
Celecoxib	—	—	—	0.04 ± 0.002	322.30
6b	4-CH_3_C_6_H_5_	—	—	0.07 ± 0.006	103.18
6c	4-ClC_6_H_5_	—	—	0.08 ± 0.006	84.00
6d	4-OHC_6_H_5_	—	—	0.05 ± 0.006	103.12
7a	—	H	H	0.05 ± 0.006	104.70
7e	—	*n*-Propyl	Cl	0.08 ± 0.006	103.60
7f	—	*n*-Propyl	Br	0.07 ± 0.006	102.27

### 8.2 *In vivo* assay of most potent compound 6d ^22^

Compound 6d exhibited the most pronounced anti-inflammatory effect, producing the greatest reduction in paw thickness at the 5 h time point (45.61% inhibition) and demonstrating the fastest onset of action, with a marked inhibition of 76.33% within the first hour compared with the carrageenan-treated group.

Furthermore, compound 6d achieved the highest suppression of paw edema, as reflected by inhibition of the percentage increase in paw weight (65.74%), outperforming all other tested compounds. Notably, it was the only compound that showed no statistically significant difference when compared with both reference drugs (Table 4).

**Table 4:** Paw thickness difference at hourly intervals (and percentage of inhibition) and the percentage of paw weight increase^a^

	Paw thickness difference at hourly intervals (mm) (percentage of inhibition (%))					Paw weight increase (%)
	1st hour	2nd hour	3rd hour	4th hour	5th hour	
Control	−0.07 ± 0.07	−0.12 ± 0.08	−0.18 ± 0.09	−0.18 ± 0.09	−0.18 ± 0.08	0.99 ± 1.59^b^
Carrageenan	1.35 ± 0.14^b^	2.63 ± 0.15^b^	3.4 ± 0.13^b^	3.78 ± 0.17^b^	4.66 ± 0.14^b^	77.46 ± 4.03^b^
Celecoxib	0.98 ± 0.11^b^ (27.39%)	1.45 ± 0.15^b^^,^^c^ (44.96%)	1.72 ± 0.09^b^^,^^c^ (49.26%)	2.01 ± 0.19^b^^,^^c^ (46.85%)	2.37 ± 0.12^b,^^c^ (49.02%)	27.5 ± 4.17^b^^,^^c^
6d	0.32 ± 0.11^b^^,^^c^^,^^d^^,^^e^ (76.33%)	1.29 ± 0.16^b^^,^^c^ (50.86%)	1.63 ± 0.14^b^^,^^c^ (52.16%)	2.05 ± 0.2^b^^,^^c^ (45.79%)	2.53 ± 0.33^b^^,^^c^ (45.61%)	26.54 ± 3.38^b^^,^^c^

a The data are presented as mean ± SD and were analyzed using two-way ANOVA (for paw thickness difference) or one-way ANOVA (for paw weight increase percentage), followed by Tukey's multiple comparisons test; *n* = 6.

b Significantly different from control group at *p* < 0.05.

c Significantly different from carrageenan group at *p* < 0.05.

d Significantly different from celecoxib group at *p* < 0.05.

e Significantly different from mefenamic acid group at *p* < 0.05.

## 9 Anti-epileptic assay

### 9.1 Anticonvulsant activity in the PTZ-induced seizure model

As shown in Table 5, PTZ administration induced generalized tonic-clonic seizures in all control mice, yielding 0% seizure protection and 80% mortality, confirming the model's severity. Pretreatment with valproic acid (300 mg kg^−1^) significantly improved seizure outcomes, producing 60% protection and reducing mortality to 30%. This finding is in line with its known anticonvulsant efficacy.^23,24^ The test compounds showed substantially greater activity. The most efficacious were 6d, 6c, and 6b, which afforded 90%, 70%, and 60% protection, respectively. Notably, 6d completely abolished mortality (0%), while 6c and 6b limited deaths to 20% (*versus* 30% in the valproate group). When expressed relative to valproate, these correspond to protective potencies of 150%, 116.7%, and 100%, respectively, highlighting their superior anticonvulsant efficacy profiles. Moderate efficacy was observed with 7a and 7e (each 50% protection, 50% mortality; 83.33% relative potency), whereas 7f was the least active (30% protection, 60% mortality; 50% relative potency). Overall, the compounds can be ranked in the terms of potency as follows: 6d > 6c > 6b > 7a = 7e > 7f.

**Table 5:** Protective efficacy, relative potency, and mortality rates of test compounds in the PTZ-induced seizure model^a^

	Protection (%)	Relative protection (%)	24 h mortality (%)
PTZ	0%	—	80%
Valproic acid	60%	—	30%
6b	60%	100%	20%
6c	70%	116.67%	20%
6d	90%	150%	0%
7a	50%	83.33%	50%
7e	50%	83.33%	50%
7f	30%	50%	60%

a Seizure protection was calculated as the percentage of animals that did not develop tonic–clonic seizures. Relative protection values were expressed as percentages compared to sodium valproate (protection test/protection valproate × 100). *n* = 10. Mortality was recorded within 24 hours after PTZ challenge. Data are expressed as percentages.

### 9.2 Evaluation of seizure onset, severity, and survival in the pilocarpine-induced convulsion model

**Fig. 8:**
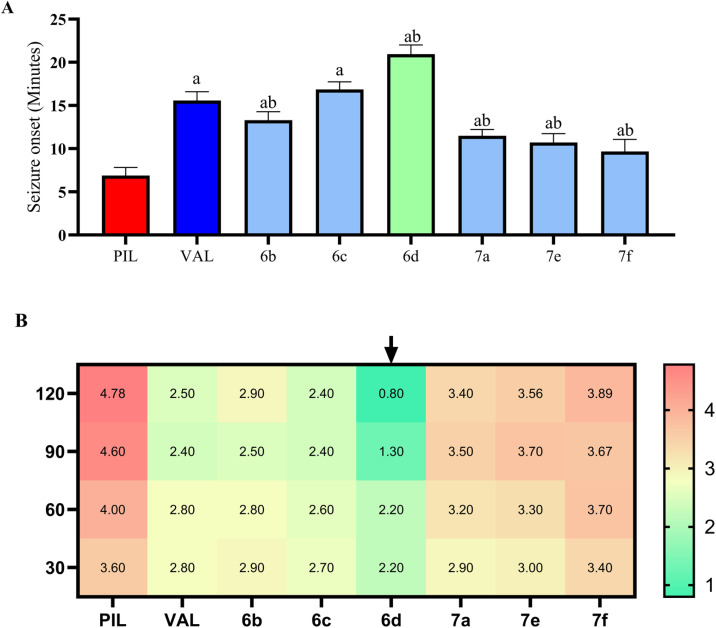
Effects of the investigated compounds on seizure onset latency and seizure intensity in the pilocarpine-induced seizure model. Panel (A) illustrates the time to onset of stage 3 seizures (in minutes) following pilocarpine administration across the different experimental groups. Panel (B) presents a heat-map visualization of seizure severity progression, expressed as Racine scores, recorded at the indicated time intervals over the 120 min monitoring period. Lower seizure intensities are depicted by green color gradients, whereas higher severity levels are indicated by darker red gradients. Data are shown as group mean values (*n* = 6), with detailed variability and statistical outcomes provided in Table 4. Seizure onset latency was analyzed using one-way ANOVA, while seizure severity over time was assessed using two-way ANOVA, followed by Tukey's post hoc multiple comparisons test. Superscript letters indicate statistically significant differences at *p* < 0.05: a, compared with the pilocarpine control group; b, compared with the valproic acid group. Abbreviations: PIL, pilocarpine control; VPA, sodium valproate.

Table 6 summarizes the outcomes of the pilocarpine model. In control mice, pilocarpine injection triggered rapid seizures: latency was only 6.89 min (6.893 ± 0.30 min, mean ± SEM) (Fig. 8A) and severity escalated to a Racine score of 4.78 by 120 min (Fig. 8B), with only 40% survival. Pretreatment with valproic acid significantly delayed seizure onset to 15.58 ± 1.01 min (an increase of 125.94% relative to control) (mean difference [MD] *vs.* pilocarpine = 8.68 min, 95% CI 7.26 to 10.10; *p* < 0.0001) (Fig. 8A) and reduced the final Racine score to 2.5 (a 47.67% decrease compared to control) (120 min: MD = 2.28 Racine units, 95% CI 1.25 to 3.31; *p* < 0.0001) (Fig. 8B). This yielded a 70% survival rate, demonstrating moderate protection.

**Table 6:** Effects of valproic acid and test compounds on seizure onset, severity, and survival in the pilocarpine-induced seizure model^a^

	Onset (min)	Seizure severity				24 h survival (%)
		30 min	60 min	90 min	120 min	
Pilocarpine	6.89 ± 0.93	3.6 ± 0.52	4 ± 0.47	4.6 ± 0.52	4.78 ± 0.44	40
Valproic acid	15.58 ± 1.01^b^	2.8 ± 0.63	2.8 ± 0.42^b^	2.4 ± 0.52^b^	2.5 ± 0.53^b^	70
6b	13.31 ± 0.97^b^^,^^c^	2.9 ± 0.32	2.8 ± 0.42^b^	2.5 ± 0.71^b^	2.9 ± 0.88^b^	80
6c	16.87 ± 0.86^b^	2.7 ± 0.67	2.6 ± 0.52^b^	2.4 ± 0.52^b^	2.4 ± 0.52^b^	80
6d	20.94 ± 1.06^b^^,^^c^	2.2 ± 0.42^b^	2.2 ± 0.42^b^	1.3 ± 0.48^b^^,^^c^	0.8 ± 0.42^b^^,^^c^	100
7a	11.5 ± 0.72^b^^,^^c^	2.9 ± 0.57	3.2 ± 0.79	3.5 ± 0.53^b^^,^^c^	3.4 ± 0.84^b^	50
7e	10.72 ± 1.03^b^^,^^c^	3 ± 0.67	3.3 ± 0.48	3.7 ± 0.67^c^	3.56 ± 0.73^b^^,^^c^	40
7f	9.67 ± 1.4^b^^,^^c^	3.4 ± 0.52	3.7 ± 0.67	3.67 ± 0.71^c^	3.89 ± 0.78^c^	40

a Data are presented as mean ± SD or percentages for survival rates. Statistical analysis was performed by one-way ANOVA for seizure onset and two-way ANOVA for seizure severity, followed by Tukey's post hoc test.

b Indicates significant difference *versus* pilocarpine group (*P* < 0.05), while.

c Indicates significance *versus* valproic acid group (*P* < 0.05).

The test compounds differed markedly. 6d was the most effective: it extended the seizure latency to 20.94 ± 1.06 min (203.82% longer than control, 34.47% longer than valproate) (*vs.* pilocarpine: MD = 14.05 min, 95% CI 12.63 to 15.47; *p* < 0.0001; *vs.* valproate: MD = 5.37 min, 95% CI 3.95 to 6.79; *p* < 0.0001) (Fig. 8A) and strongly suppressed seizure progression. At 120 min, its Racine score was only 0.8 ± 0.42 (about an 83.26% reduction *vs.* control, *versus* 47.67% for valproate) (120 min *vs.* pilocarpine: MD = 3.98 Racine units, 95% CI 2.95 to 5.01; *p* < 0.0001; *vs.* valproate: MD = 1.70, 95% CI 0.70 to 2.70; *p* < 0.0001) (Fig. 8B). Notably, 6d ensured 100% survival. Compound 6c also provided robust protection: onset was delayed to 16.87 ± 0.86 min (+144.71% *vs.* control) (*vs.* pilocarpine: MD = 9.98 min, 95% CI 8.56 to 11.39; *p* < 0.0001; *vs.* valproate: MD = 1.29 min, 95% CI −0.12 to 2.71; *p* = 0.0991), final severity was 2.4 ± 0.52 (49.77% reduction) (120 min *vs.* pilocarpine: MD = 2.38 Racine units, 95% CI 1.35 to 3.41; *p* < 0.0001; *vs.* valproate: MD = 0.10, 95% CI −0.90 to 1.10; *p* > 0.9999), and 80% of mice survived. 6b showed intermediate activity: latency 13.31 ± 0.97 min (+93.09%) (*vs.* pilocarpine: MD = 6.42 min, 95% CI 5.00 to 7.83; *p* < 0.0001; *vs.* valproate: MD = −2.27 min, 95% CI −3.68 to −0.85; *p* = 0.0001), final score 2.9 ± 0.88 (39.3% reduction) (120 min *vs.* pilocarpine: MD = 1.88 Racine units, 95% CI 0.85 to 2.91; *p* < 0.0001; *vs.* valproate: MD = −0.40, 95% CI −1.40 to 0.60; *p* = 0.9998), and 80% survival. In contrast, 7a, 7e, and 7f were less efficacious. They produced smaller delays in onset (on the order of +40–67% *vs.* control) (all significantly longer than pilocarpine, *p* < 0.0001) and only partial seizure control. Their final Racine scores remained high (>3.4), and survival was only 40–50%. In fact, 7e and 7f showed significantly higher severity scores than the valproate group at later time points, indicating lower protection (7e*vs.* valproate: 90 min, MD = 1.30, 95% CI 0.30 to 2.30, *p* = 0.0006; 120 min, MD = 1.06, 95% CI 0.03 to 2.09, *p* = 0.0364; 7f*vs.* valproate: 90 min, MD = 1.27, 95% CI 0.24 to 2.30, *p* = 0.0017; 120 min, MD = 1.39, 95% CI 0.36 to 2.42, *p* = 0.0002).

Overall, 6d clearly outperformed valproate and the other compounds in the pilocarpine model. It not only delayed seizures and reduced their severity to near-normal levels, but also prevented mortality. Its superiority over valproate was statistically significant for seizure latency and for seizure severity at 90- and 120 min. Compound 6c performed comparably to valproate in many respects, with no significant difference from valproate in seizure latency (adjusted *p* = 0.0991) or final Racine score at 120 min (*p* > 0.9999), and 6b was somewhat less effective but still markedly better than the other compounds, 7a, 7e, and 7f. These results underscore 6d > 6c > 6b as the leading candidates, with 6d showing a multi-fold benefit over valproate in latency extension and seizure suppression.

### 9.3 Structure–activity relationship analysis based on *in vivo* anticonvulsant models

#### 9.3.1 Impact of phenyl ring substitution (series 6)

**Fig. 9:**
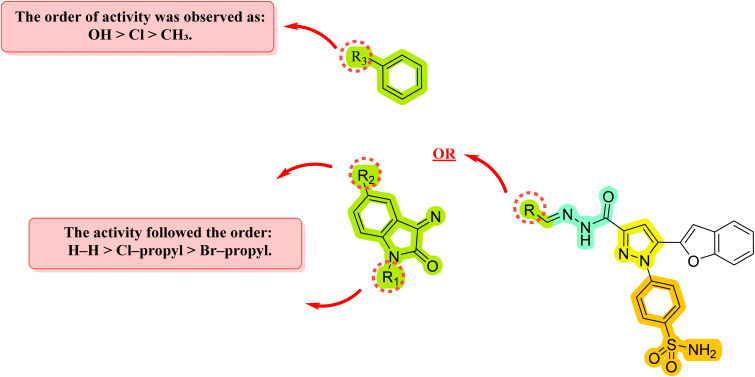
SAR investigation of the designed compounds 6b–d, 7a, 7e, and 7f in different *in vivo* seizure models.

• Electronic effects: the introduction of a methyl group 6b at the phenyl ring, which exerts a positive inductive effect, resulted in a reduction in potency. This suggests that increasing electron density on the aryl moiety may be unfavorable for target binding.

• Halogenation: conversely, substitution with a chloro atom 6c, which possesses a strong negative inductive effect and lipophilic character, led to a marked enhancement in activity. This improvement likely stems from improved hydrophobic interactions within the binding pocket.

• Hydrogen-bonding potential: the highest anticonvulsant efficacy was achieved with the hydroxyl-substituted analog 6d. This indicates that the presence of a strong hydrogen-bond donor (HBD) is a critical structural requirement, potentially facilitating key dipole–dipole interactions with the biological target that stabilize the ligand–receptor complex (Fig. 9).

#### 9.3.2 Evaluation of the isatin scaffold (series 7)

• Scaffold morphing: replacement of the phenyl moiety with a more rigid, bicyclic isatin (indoline-2,3-dione) scaffold 7a, 7e, and 7f yielded a distinct SAR trend. The unsubstituted isatin derivative 7a maintained moderate activity, highlighting the bioisosteric potential of the indole-based system.

• *N*-alkylation and halogenation: interestingly, the combination of halogen substitution (chloro or bromo) on the isatin nucleus with *N*-alkylation *via* a propyl group (7e and 7f) resulted in a significant loss of potency (Fig. 9).

### 9.4 Impact of test compounds on pilocarpine-induced hippocampal alterations

Oxidative stress and neuroinflammation are key pathological drivers of seizure progression and neuronal injury. Seizure activity is associated with excessive generation of reactive oxygen and nitrogen species, which destabilize neuronal membranes and mitochondria, leading to increased lipid peroxidation and nitric oxide production.^25–27^ At the same time, excessive glutamatergic neurotransmission amplifies excitotoxicity and overwhelms endogenous antioxidant defenses.^28,29^ Neuroinflammatory cascades further contribute to neuronal damage by upregulating cytokines such as TNF-α and IL-6, which perpetuate injury.^30–32^ These interdependent mechanisms underscore the importance of developing therapeutic strategies that simultaneously counter oxidative stress, excitotoxicity, and inflammation. Based on its superior anticonvulsant profile, compound 6d was selected for detailed biochemical analysis in hippocampal tissue.

**Fig. 10:**
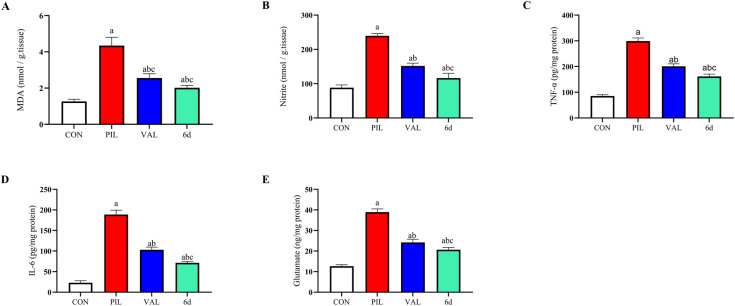
Effects of experimental compound 6d on hippocampal oxidative stress, inflammation, and excitotoxicity markers. Data (mean ± SD, *n* = 6) were analyzed using one-way ANOVA, followed by Tukey's post hoc test for multiple comparisons. Statistically significant differences are marked as: ^a^ for comparisons with the normal control group (*P* < 0.05), ^b^ for comparisons with the pilocarpine group (*P* < 0.05), and ^c^ for comparisons with the valproic acid group (*P* < 0.05). Abbreviations: CON, normal control; PIL, pilocarpine control; VPA, sodium valproate.

As shown in Fig. 10, hippocampal MDA and nitrite (markers of lipid peroxidation and nitrosative stress) were dramatically elevated after pilocarpine (MDA: +242.86%; nitrite: +171.38%) (CON *vs.* PIL: MDA MD = −3.075, 95% CI −3.517 to −2.633, *p* < 0.0001; nitrite MD = −151.3, 95% CI −167.0 to −135.6, *p* < 0.0001). Valproic acid significantly blunted this oxidative surge: MDA was reduced by 41.07% and nitrite by 36.72% compared to the pilocarpine group. Compound 6d produced even greater antioxidant effects. 6d lowered MDA by 53.57% *vs.* pilocarpine and nitrite by 51.48% (PIL *vs.*6d: MDA MD = 2.326, 95% CI 1.883 to 2.768, *p* < 0.0001; nitrite MD = 123.3, 95% CI 107.7 to 139.0, *p* < 0.0001). Relative to valproate, these represent additional improvements of 21.21% (MDA) and 23.31% (nitrite) (VAL *vs.*6d: MDA MD = 0.5426, 95% CI 0.1003 to 0.9849, *p* = 0.0129; nitrite MD = 35.35, 95% CI 19.66 to 51.03, *p* < 0.0001). Thus, 6d more effectively neutralized seizure-induced oxidative damage than valproate.

Pilocarpine also greatly upregulated hippocampal inflammatory markers. TNF-α rose 250.09%, IL-6 by 717.04%, and glutamate by 207.81% relative to controls (CON *vs.* PIL: TNF-α MD = −213.8, 95% CI -228.9 to −198.6, *p* < 0.0001; IL-6 MD = −165.7, 95% CI −176.6 to −154.9, *p* < 0.0001; glutamate MD = −26.30, 95% CI −28.32 to −24.27, *p* < 0.0001). Valproate pretreatment significantly attenuated these increases (TNF-α by 32.89%, IL-6 by 45.44%, and glutamate by 37.85% *vs.* pilocarpine) (PIL *vs.* VAL: TNF-α MD = 98.44, 95% CI 83.30 to 113.6, *p* < 0.0001; IL-6 MD = 85.82, 95% CI 74.99 to 96.66, *p* < 0.0001; glutamate MD = 14.74, 95% CI 12.71 to 16.77, *p* < 0.0001). Again, 6d outperformed valproate on all measures. With 6d, significantly lowering TNF-α by 46.03% from pilocarpine (19.57% further reduction beyond valproate), IL-6 by 62.25% relative to pilocarpine (30.81% extra to valproate), and glutamate by 46.99% *vs.* pilocarpine (14.7% *vs.* valproate) (PIL *vs.*6d: TNF-α MD = 137.7, 95% CI 122.6 to 152.9, *p* < 0.0001; IL-6 MD = 117.6, 95% CI 106.7 to 128.4, *p* < 0.0001; glutamate MD = 18.30, 95% CI 16.27 to 20.33, *p* < 0.0001; VAL *vs.*6d: TNF-α MD = 39.30, 95% CI 24.16 to 54.44, *p* < 0.0001; IL-6 MD = 31.75, 95% CI 20.92 to 42.59, *p* < 0.0001; glutamate MD = 3.558, 95% CI 1.529 to 5.586, *p* = 0.0005). In practical terms, 6d brought cytokines and excitatory glutamate much closer to control levels than valproate did. These data demonstrate that 6d exerts strong anti-inflammatory and anti-excitotoxic actions in the hippocampus.

The epileptogenic process is driven by a complex interplay between oxidative stress, excitotoxic neurotransmission, and inflammatory signaling within vulnerable brain regions. In the present investigation, pilocarpine challenge elicited a marked increase in hippocampal oxidative burden, as evidenced by significant elevations in MDA and nitrite levels. MDA, a well-recognized indicator of seizure-associated lipid peroxidation,^25,27^ reflects extensive oxidative damage to neuronal membranes, while the concomitant rise in nitrite suggests excessive nitric oxide production and subsequent formation of reactive nitrogen species, including peroxynitrite, which are known to impair mitochondrial function and promote neuronal loss.^33^ Pretreatment with compound 6d markedly attenuated these oxidative alterations, indicating a pronounced antioxidant capacity that may contribute to preservation of neuronal integrity and mitigation of free radical-mediated neurotoxicity.

Beyond oxidative stress, pilocarpine-induced seizures were accompanied by a substantial enhancement of glutamatergic activity, a central feature of excitotoxic injury. Pathological accumulation of extracellular glutamate leads to sustained activation of postsynaptic glutamate receptors, resulting in excessive calcium influx, mitochondrial dysfunction, and progressive neuronal degeneration.^34^ The test compound 6d significantly reduced hippocampal glutamate levels. This modulation of glutamatergic signaling suggests an important role in restoring the excitatory-inhibitory balance and limiting glutamate-driven excitotoxic cascades.

In parallel, pilocarpine provoked a pronounced neuroinflammatory response, characterized by marked upregulation of the proinflammatory cytokines TNF-α and IL-6. These mediators are critically involved in blood–brain barrier dysfunction, enhanced neuronal excitability, and the maintenance of recurrent seizure activity.^30^ Pretreatment with compound 6d significantly suppressed the pilocarpine-induced cytokine surge. Collectively, these findings underscore the ability of compound 6d to counteract oxidative stress, glutamate-mediated excitotoxicity, and neuroinflammation, three interrelated processes that drive epileptogenesis.

The reduction in pro-inflammatory cytokines such as TNF-α and IL-6 observed after treatment with compound 6d suggests suppression of neuroinflammatory signaling in hippocampal tissue. Neuroinflammation is known to enhance neuronal excitability and facilitate seizure propagation. Therefore, attenuation of these inflammatory mediators may contribute to the delayed seizure onset and reduced seizure severity observed in both PTZ and pilocarpine models.

### 9.5 Toxicity and safety evaluation

A comprehensive serum biochemical panel was performed to evaluate the subchronic systemic safety of the lead compound 6d following once-daily oral administration for 14 consecutive days. Across all evaluated parameters, biochemical values remained within physiological limits and did not differ significantly from those of the normal control group, indicating good overall tolerability under the applied dosing conditions (Table 7).

**Table 7:** Toxicological biochemical parameters for liver, kidney, and cardiac function^a^

	ALT (IU L^−1^)	AST (IU L^−1^)	Serum urea (mg dL^−1^)	Serum creatinine (mg dL^−1^)	CK-MB (Pg mL^−1^)	Troponin T (Pg mL^−1^)
Control	76.16 ± 5.27	88.22 ± 3.04	0.45 ± 0.02	21.69 ± 1.15	5.49 ± 0.31	20.66 ± 0.82
6d	78.12 ± 8.53	83.98 ± 4.73	0.46 ± 0.03	22.82 ± 1.57	5.4 ± 0.26	19.32 ± 1.61

a Data (mean ± SD, *n* = 6) were analyzed using unpaired Student's *t*-test. No statistically significant differences were observed across the groups for any of the evaluated parameters (*P* > 0.05).

Assessment of hepatic safety was based on the determination of serum alanine aminotransferase (ALT) and aspartate aminotransferase (AST), which are widely accepted indicators of hepatocellular membrane integrity and enzyme leakage. Subchronic exposure to compound 6d did not produce any significant alterations in ALT or AST activities compared with control animals, suggesting preservation of normal liver function at the tested dose. These findings are particularly relevant given the established sensitivity of these enzymes as early biomarkers of drug-induced hepatic stress in preclinical investigations.^35,36^

Renal safety was assessed by measuring serum urea and creatinine concentrations, parameters that reflect glomerular filtration efficiency and renal handling capacity. Following 14 days of treatment with 6d, both markers remained comparable to control values and showed no statistically significant changes. This finding indicates that subchronic administration of the compound did not adversely affect renal function, in agreement with previous reports highlighting the reliability of urea and creatinine as indicators of nephrotoxic liability in animal studies.^37,38^

Potential cardiotoxicity was further investigated by quantifying serum creatine kinase-MB (CK-MB) and troponin T, two biomarkers closely associated with myocardial injury. Levels of CK-MB remained stable following repeated dosing with compound 6d, and troponin T concentrations similarly showed no evidence of cardiac insult. Given the established utility of these biomarkers in early detection of compound-induced cardiotoxicity, their unaltered levels support the absence of detectable adverse cardiac effects under the present experimental conditions.^39–42^

Collectively, the lack of significant changes in hepatic, renal, and cardiac biochemical indices following 14 days of subchronic administration indicates that compound 6d exhibits a favorable preliminary safety profile *in vivo*. When considered alongside its pronounced pharmacological efficacy in anticonvulsant models, these data support the advancement of 6d for further development and justify more extensive safety and toxicological evaluations in future studies.

## 10 Molecular docking study

Molecular docking studies were performed within the active sites of voltage-gated calcium channels (VGCCs), (PDB ID: 2COJ). The docking grid was centered at coordinates *x* = −36.14, *y* = −6.293, *z* = −17.866 to encompass the active site. Docking calculations were conducted using the Autodock Vina software scoring function. The docking protocol was validated using gabapentin ([1-(aminomethyl)cyclohexyl]acetic acid) as a reference ligand, with redocking producing RMSD values of 0 Å for both upper and lower limits, confirming the reliability of the protocol (Fig. S2).

Subsequently, compound 6d was docked to assess its ability to mimic or enhance the binding interactions of the reference drug and the antiepileptic drug valproic acid (VI), nine binding poses were generated, and the best pose was selected, yielding a best docking score of −10.3 kcal mol^−1^. The comparative docking results supported the anticonvulsant potential of compound 6d and provide a hypothesis for the potential anticonvulsant mechanism, suggesting possible modulation of calcium channel activity.

**Fig. 11:**
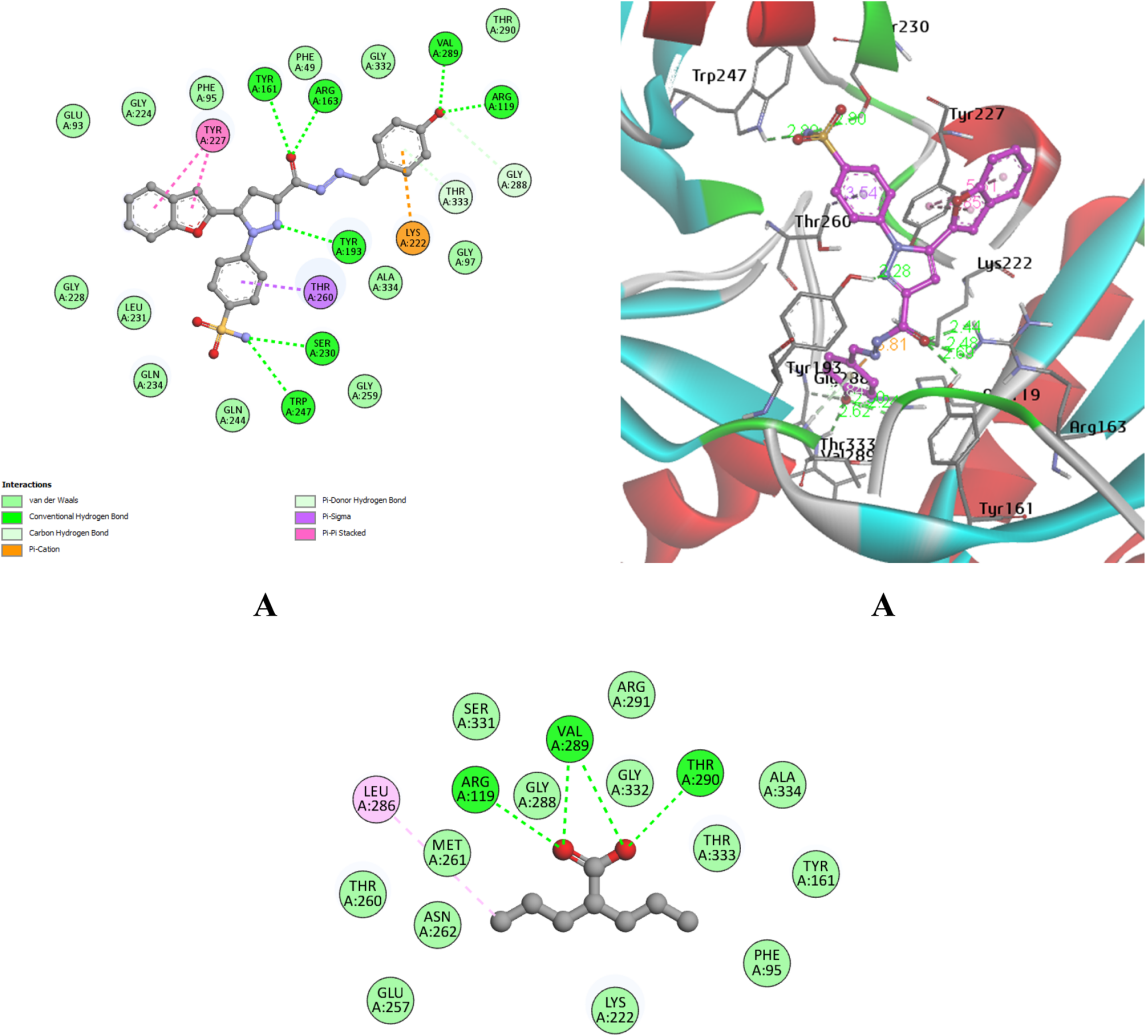
2D and 3D schematic representations of compound 6d bound within the active sites of (VGCCs) (A) compared with the binding mode of the reference drug valproic acid.

The reference drug, valproic acid, was found to form hydrogen-bonding interactions *via* its carboxylate moiety with key amino acid residues in the active site of the voltage-gated sodium/calcium channel, including Thr29, Val289, and Arg119. In a comparable manner, the most potent compound 6d established hydrogen-bond interactions with Arg119 and Val289. Additionally, the hydrazide linker of compound 6d interacted with Tyr161 and Arg163 *via* its carbonyl group, while an additional hydrogen bond was formed with Tyr193 through the pyrazole moiety. The benzofuran moiety engaged in π–π stacking interactions with Tyr227. Finally, the sulfonamide group formed hydrogen bonds with Ser230 and Trp247, thereby stabilizing the ligand within the binding pocket (Fig. 11).

## 11 Conclusion

Neuroinflammation, oxidative stress, and glutamate-mediated excitotoxicity are recognized as key pathological correlates of epileptogenesis and seizure propagation. In the present study, compound 6d emerged as a potent anticonvulsant lead with a pronounced multimodal neuroprotective profile. *In vivo* evaluations demonstrated superior efficacy; 6d afforded 90% protection in the pentylenetetrazol (PTZ) model and significantly delayed seizure onset while attenuating severity in the pilocarpine-induced model, achieving 100% survival.

Parallel to its behavioral effects, compound 6d elicited significant biochemical shifts within hippocampal tissue. Treatment led to a marked reduction in oxidative stress markers (MDA and nitrite), pro-inflammatory cytokines (TNF-alpha and IL-6), and glutamate levels. While these results indicate a suppression of neuroinflammatory and excitotoxic pathways, these biochemical changes should be interpreted as supportive evidence of the compound's broad neuroprotective influence rather than a demonstrated causal mechanism for its antiseizure activity. The robust pharmacological profile of 6d may be partially attributed to its hydroxyl substituent, which molecular modeling suggests facilitates essential hydrogen-bonding interactions within the target binding pocket. These interactions likely stabilize the ligand–target complex, potentially contributing to both the termination of seizure activity and the concomitant modulation of downstream inflammatory markers.

Furthermore, repeated oral administration of 6d revealed a favorable preliminary safety profile, with no detectable toxicity in hepatic, renal, or cardiac tissues. In summary, while 6d robustly attenuates hippocampal neuroinflammation and oxidative stress in tandem with its anticonvulsant action, further investigation is warranted to delineate the specific mechanistic hierarchy and determine whether these anti-inflammatory effects are a primary driver or a secondary consequence of seizure suppression.

Within the SAR framework, compound 6d demonstrated a marked enhancement in anticonvulsant potency and neuroprotective efficacy compared to its analogues, suggesting a critical role of its specific structural features. In particular, the presence of the hydroxyl substituent appears to be a key determinant of activity, potentially enabling additional hydrogen-bonding interactions within the target binding site, as supported by molecular modeling analysis. This structural attribute may contribute to improved target affinity and, consequently, superior biological performance. Furthermore, the ability of compound 6d to significantly reduce oxidative stress markers, pro-inflammatory cytokines, and glutamate levels indicates that such structural modifications not only enhance anticonvulsant activity but also promote modulation of neuroinflammatory and excitotoxic pathways.

## 12 Experimental

### 12.1 Synthesis of pyrazole sulfonamide 6b–d, 7a, 7e, and 7f (ref. 22)

Compound 2, ethyl 4-(benzofuran-2-yl)-2,4-dioxobutanoate, was reacted with 4-aminosulfonylphenylhydrazine in an ethanol/acetic acid medium to give ethyl 5-(benzofuran-2-yl)-1-(4-sulfamoylphenyl)-1*H*-pyrazole-3-carboxylate 3. Treatment of ester 3 with hydrazine hydrate under reflux conditions afforded the corresponding hydrazide derivative, 4-(5-(benzofuran-2-yl)-3-(hydrazinecarbonyl)-1*H*-pyrazol-1-yl)benzenesulfonamide 4. Further refluxing of compound 4 underwent condensation with a series of substituted aldehydes or isatins in refluxing ethanol containing a catalytic amount of acetic acid, leading to the formation of the target compounds 6b–d and 7a, 7e, 7f (SI).

The HPLC purity results were included in the SI file.

### 12.2 *In silico* and biological studies^43–45^

Detailed methodologies for molecular docking, and bioinformatics analyses, together with the experimental protocols for the *in vivo* anti-inflammatory, anticonvulsant, and analgesic models, ELISA measurements, and toxicological studies, are described in the SI.

## 13 Limitations of the study

Despite the promising findings, several limitations should be acknowledged. First, the *in vivo* anticonvulsant models employed in this study, while widely accepted, may not fully recapitulate the complexity and heterogeneity of human epilepsy, which could limit the direct translational relevance of the results. Second, the molecular docking analysis provides only a static representation of ligand–target interactions and does not account for protein flexibility, solvent effects, or dynamic conformational changes, which may influence binding affinity and accuracy of the predicted interactions. In addition, the sample size used in the biological evaluation was relatively limited, which may affect the statistical robustness of the observed effects. Furthermore, although the designed compounds demonstrated promising anticonvulsant activity, potential off-target effects and long-term safety profiles were not fully explored in the current study.

## Conflicts of interest

The authors report no conflicts of interest related to this work.

## Supplementary Material

RA-016-D6RA01165A-s001

## Data Availability

The data supporting this article have been included as part of the supplementary information (SI). Supplementary information is available. See DOI: https://doi.org/10.1039/d6ra01165a.
