# Supplementary material for: From molecular design to antiepileptic evaluation: sulfonamide–pyrazole derivatives as promising neuroactive agents
Source: RSC Adv. 2026 May 26;16(31):28667–84. doi: 10.1039/d6ra01165a (PMC13213577; doi:10.1039/d6ra01165a)
Supplement: RA-016-D6RA01165A-s001 [file RA-016-D6RA01165A-s001.pdf]

Supplementary Information (SI) for RSC Advances.
This journal is © The Royal Society of Chemistry 2026

Supplementary data

From Molecular Design to Antiepileptic Evaluation: Sulfonamide–Pyrazole Derivatives as Promising Neuroactive Agents

Mohamed K. Elgohary[a, *], Mahmoud Abdelrahman Alkabbani[b], Aya Mohamed Ahmed Ibrahim[c], Ahmed Elsonbaty[a], Abdelhameed Abubakr[d], Mayada H. Mohamed[e], Abdulrahman A. Almehizia[f], Ahmed M. Naglah[f*], Mohamed Fares [a, g], Hatem A. Abdel-Aziz[h]

[a] *Pharmaceutical Chemistry Department, Faculty of Pharmacy, Egyptian Russian University, Badr City, Cairo, 11829, Egypt*

[b] *Pharmacology and Toxicology Department, Faculty of Pharmacy, Egyptian-Russian University, Badr City, Cairo, 11829, Egypt*

[c] *Department of Biochemistry, Faculty of Pharmacy, Egyptian Russian University, Badr City, Cairo, Egypt.*

[d] *Department of Pharmaceutics and Pharmaceutical Technology, Egyptian Russian University, Badr City, Cairo, Egypt.*

[e] *University Family Medicine Center, Department of Family and Community Medicine, College of Medicine, King Saud University Medical City, P.O. Box 2925, Riyadh 11472, Saudi Arabia.*

[f] *Drug Exploration and Development Chair (DEDC), Department of Pharmaceutical Chemistry, College of Pharmacy, King Saud University, P.O. Box 2457, Riyadh 11451, Saudi Arabia.*

[g] *School of Pharmacy, The University of Sydney, Sydney, NSW, 2006, Australia*

[h] *Applied Organic Chemistry Department, National Research Center, Dokki, Cairo, 12622, Egypt*

Corresponding author: mohamed-elgohary@eru.edu.eg, anaglah@ksu.edu.sa,

Toxicity impact

ProTox-3.0 - Prediction of TOXicity of chemicals

Classification	Target	Shorthand	Prediction	Probability
Organ toxicity	Hepatotoxicity	dili	Inactive	0.50
Organ toxicity	Neurotoxicity	neuro	Inactive	0.72
Organ toxicity	Nephrotoxicity	nephro	Active	0.50
Organ toxicity	Respiratory toxicity	respi	Active	0.58
Organ toxicity	Cardiotoxicity	cardio	Inactive	0.79
Toxicity end points	Carcinogenicity	carcino	Inactive	0.50
Toxicity end points	Immunotoxicity	immuno	Inactive	0.65
Toxicity end points	Mutagenicity	mutagen	Inactive	0.64
Toxicity end points	Cytotoxicity	cyto	Inactive	0.67
Toxicity end points	BBB-barrier	bbb	Inactive	0.57
Toxicity end points	Ecotoxicity	eco	Inactive	0.78
Toxicity end points	Clinical toxicity	clinical	Active	0.51
Toxicity end points	Nutritional toxicity	nutri	Inactive	0.58
Tox21-Nuclear receptor signalling pathways	Aryl hydrocarbon Receptor (AhR)	nr_ahr	Inactive	0.93
Tox21-Nuclear receptor signalling pathways	Androgen Receptor (AR)	nr_ar	Inactive	0.97
Tox21-Nuclear receptor signalling pathways	Androgen Receptor Ligand Binding Domain (AR-LBD)	nr_ar_lbd	Inactive	0.99
Tox21-Nuclear receptor signalling pathways	Aromatase	nr_aromatase	Inactive	0.97
Tox21-Nuclear receptor signalling pathways	Estrogen Receptor Alpha (ER)	nr_er	Inactive	0.92
Tox21-Nuclear receptor signalling pathways	Estrogen Receptor Ligand Binding Domain (ER-LBD)	nr_er_lbd	Inactive	0.98
Tox21-Nuclear receptor signalling pathways	Peroxisome Proliferator Activated Receptor Gamma (PPAR-Gamma)	nr_ppar_gamma	Inactive	0.95
Tox21-Stress response pathways	Nuclear factor (erythroid-derived 2)-like 2/ antioxidant responsive element (nrf2/ARE)	sr_are	Inactive	0.97
Tox21-Stress response pathways	Heat shock factor response element (HSE)	sr_hse	Inactive	0.97
Tox21-Stress response pathways	Mitochondrial Membrane Potential (MMP)	sr_mmp	Inactive	0.74
Tox21-Stress response pathways	Phosphoprotein (Tumor Supressor) p53	sr_p53	Inactive	0.93
Tox21-Stress response pathways	ATPase family AAA domain-containing protein 5 (ATAD5)	sr_atad5	Inactive	0.98
Molecular Initiating Events	Thyroid hormone receptor alpha (THRα)	mie_thr_alpha	Inactive	0.83
Molecular Initiating Events	Thyroid hormone receptor beta (THRβ)	mie_thr_beta	Inactive	0.83
Molecular Initiating Events	Transtyretrin (TTR)	mie_ttr	Inactive	0.63
Molecular Initiating Events	Ryanodine receptor (RYR)	mie_ryr	Inactive	0.87
Molecular Initiating Events	GABA receptor (GABAR)	mie_gabar	Inactive	0.95
Molecular Initiating Events	Glutamate N-methyl-D-aspartate receptor (NMDAR)	mie_nmdar	Inactive	0.98
Molecular Initiating	alpha-amino-3-hydroxy-5-methyl-4-	mie_ampar	Inactive	0.85

Classification	Target	Shorthand	Prediction	Probability
Events	isoxazolepropionate receptor (AMPAR)			
Molecular Initiating Events	Kainate receptor (KAR)	mie_kar	Inactive	0.96
Molecular Initiating Events	Achetylcholinesterase (AChE)	mie_ache	Inactive	0.96
Molecular Initiating Events	Constitutive androstane receptor (CAR)	mie_car	Inactive	1.0
Molecular Initiating Events	Pregnane X receptor (PXR)	mie_pxr	Inactive	0.61
Molecular Initiating Events	NADH-quinone oxidoreductase (NADHOX)	mie_nadhox	Inactive	0.98
Molecular Initiating Events	Voltage gated sodium channel (VGSC)	mie_vgsc	Inactive	0.52
Molecular Initiating Events	Na+/I- symporter (NIS)	mie_nis	Inactive	0.95
Metabolism	Cytochrome CYP1A2	CYP1A2	Inactive	0.97
Metabolism	Cytochrome CYP2C19	CYP2C19	Inactive	0.93
Metabolism	Cytochrome CYP2C9	CYP2C9	Inactive	0.52
Metabolism	Cytochrome CYP2D6	CYP2D6	Inactive	0.92
Metabolism	Cytochrome CYP3A4	CYP3A4	Inactive	0.91
Metabolism	Cytochrome CYP2E1	CYP2E1	Inactive	1.0

Figure S1: *Toxicity impact of compound* ***6d*** *using ProTox 3.0 program.*

Molecular docking survey

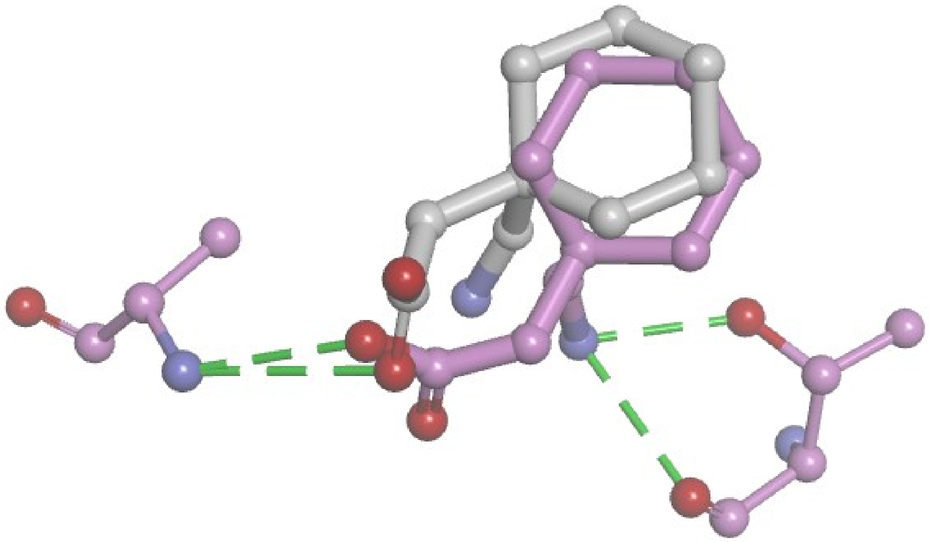

```
mode |   affinity | dist from best mode
     | (kcal/mol) | rmsd l.b.| rmsd u.b.
-----+------------+----------+----------
   1         -5.5      0.000      0.000
   2         -5.2      2.142      3.012
   3         -5.0     11.938     13.072
   4         -4.9      1.961      2.784
   5         -4.9      2.377      3.152
   6         -4.8     11.793     12.482
   7         -4.8      1.952      2.452
   8         -4.7      2.794      3.591
   9         -4.7      8.714     10.064
Writing output ... done.
```

Figure S2: *Superimposition of co-crystalized gabapentin (purple) and redocked gabapentin (white) for validation of docking protocol.*

Experimental sections

1 Chemistry

1.1 Synthesis of ethyl 5-(benzofuran-2-yl)-1-(4-sulfamoylphenyl)-1H-pyrazole-3-carboxylate (3). [1]

Ethyl 4-(benzofuran-2-yl)-2,4-dioxobutanoate **2** [2] (4 mmol, 0.88 g) was dissolved in 15 mL of acetic acid, followed by the addition of a solution of 4-aminosulfonylphenylhydrazine (4 mmol, 0.75 g) in 20 mL of EtOH. The reaction mixture was then refluxed for 3 h. Upon completion, the resulting precipitate was filtered, dried, and recrystallized from ethanol to afford compound **3**.

1.2 Synthesis of 4-(5-(benzofuran-2-yl)-3-(hydrazinecarbonyl)-1H-pyrazol-1-yl)benzenesulfonamide (4).

Ester **3** (10 mmol, 3.71 g) was refluxed in 10 mL of hydrazine hydrate and the reaction progress was monitored by TLC. After 3 h, upon completion of the reaction, the mixture was poured over ice and stirred for 3 hr, with a few drops of acetic acid added. The resulting precipitate **4** was filtered, dried, and recrystallized from EtOH.

1.3 Synthesis of 2-(5-(benzofuran-2-yl)-1-(4-sulfamoylphenyl)-1H-pyrazole-3-carbonyl)-N-phenylhydrazine-1-carboxamide (5)

Hydrazide compound **4** (1 mmol, 0.36 g) was refluxed in 10 mL of EtOH in the presence of phenyl isothiocyanate, with the progress of the reaction monitored by TLC. After 1 hr, upon completion of the reaction, the mixture was filtered and subsequently dried to yield tilted compound **5**.

1.4 Synthesis the final compounds (6a-e, 7a-f)

A solution of hydrazide **4** (1 mmol, 0.36 g) in 20 mL of ethanol was combined with the appropriatealdehydes or isatin (1 mmol) and a catalytic amount of acetic acid (0.5 mL). The mixture was then refluxed for 6 h. Upon completion, the precipitate that formed was filtered, and recrystallized from a EtOH to yield the target compounds **6a-e** and **7a-f**.

HPLC charts

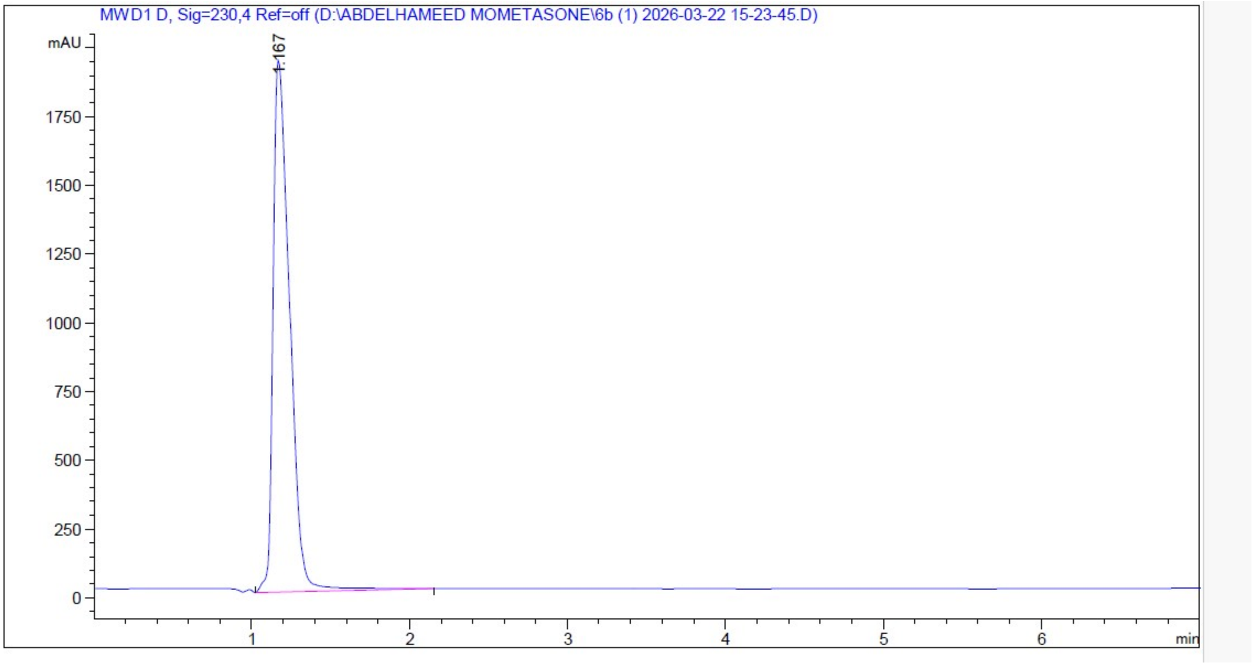

*HPLC chart of compound **6b***

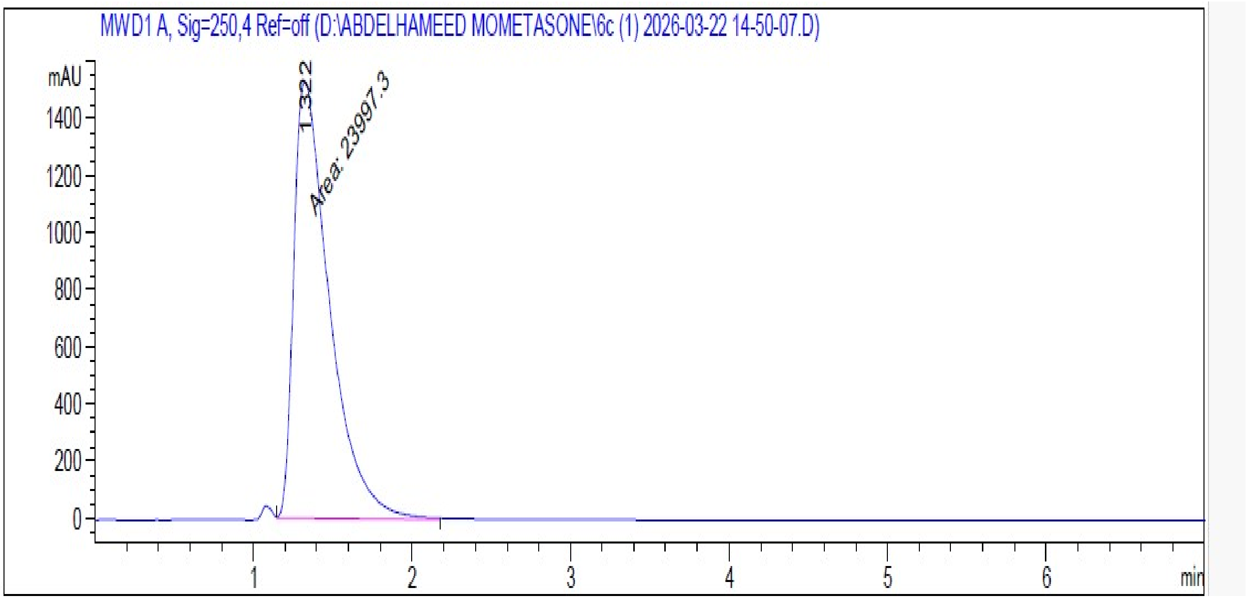

*HPLC chart of compound **6c***

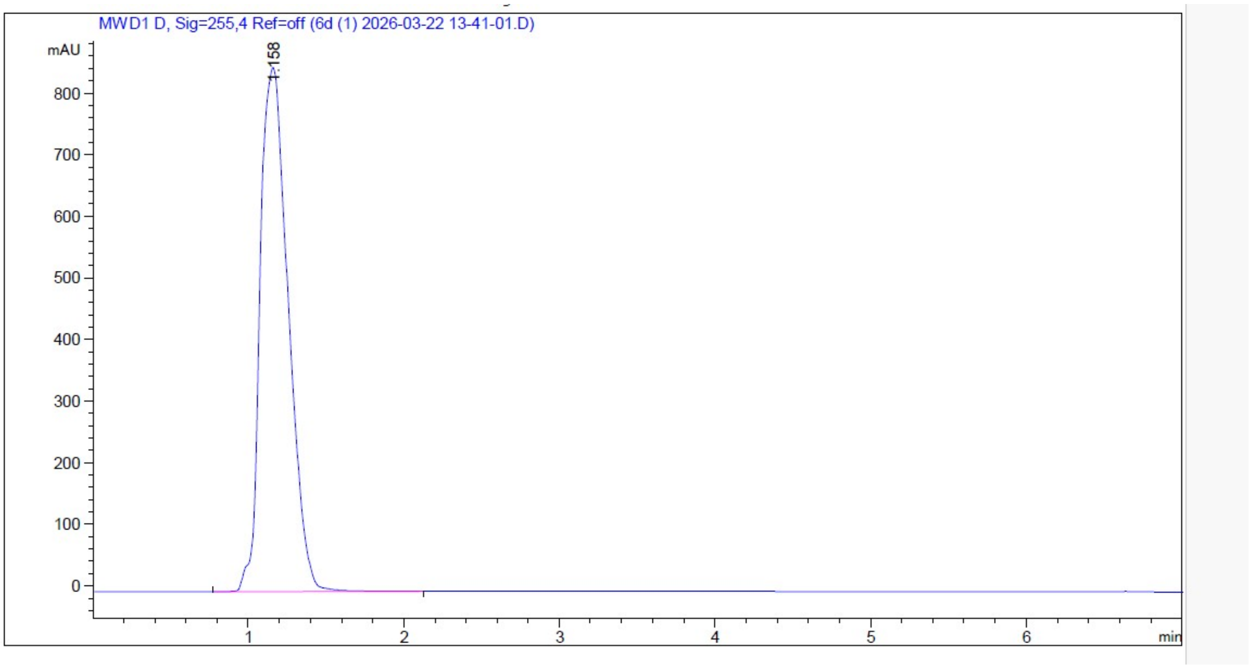

*<u>HPLC chart of compound **6d**</u>*

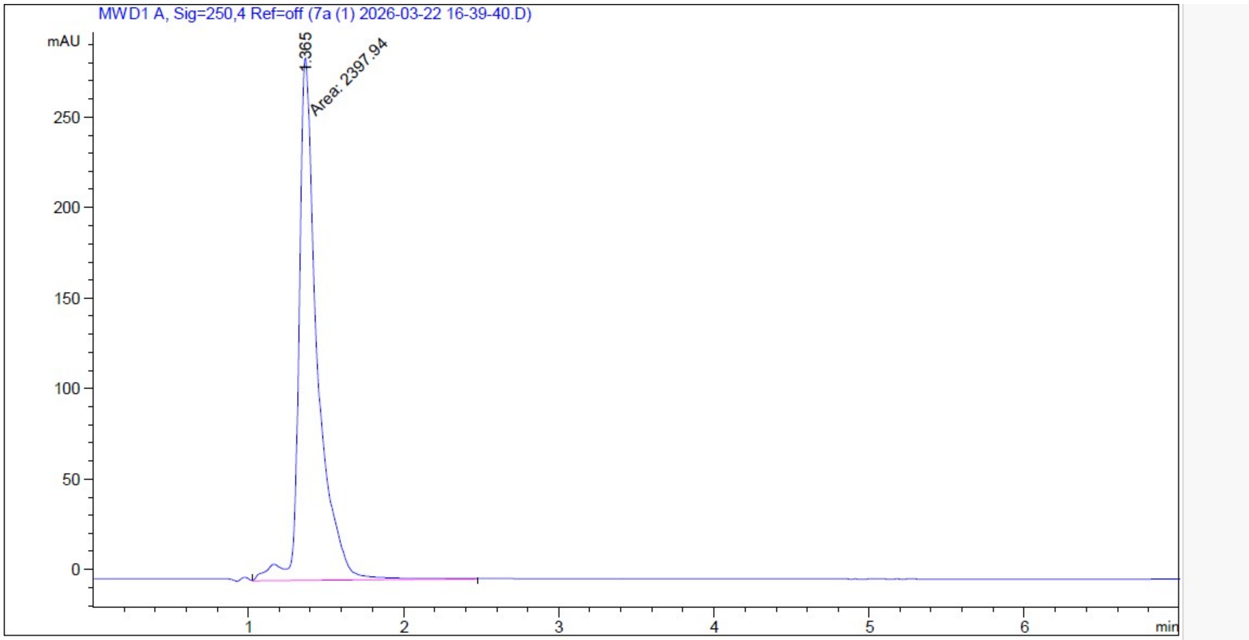

*<u>HPLC chart of compound 7**a**</u>*

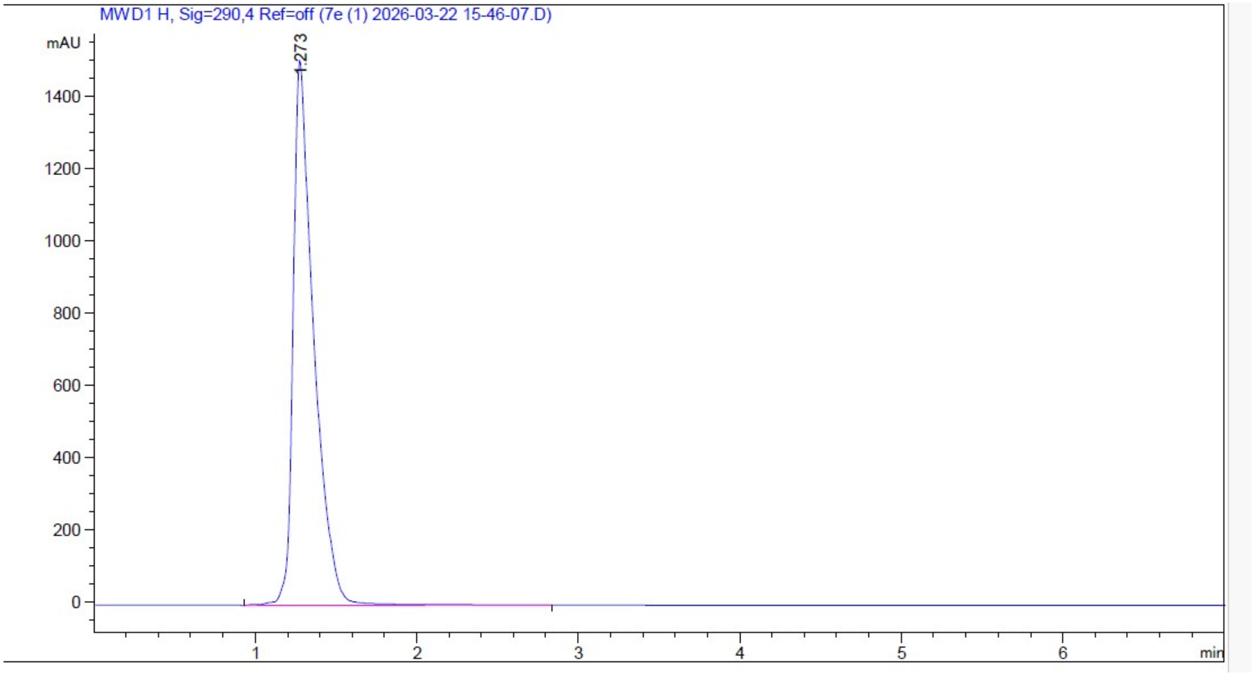

*HPLC chart of compound 7**e***

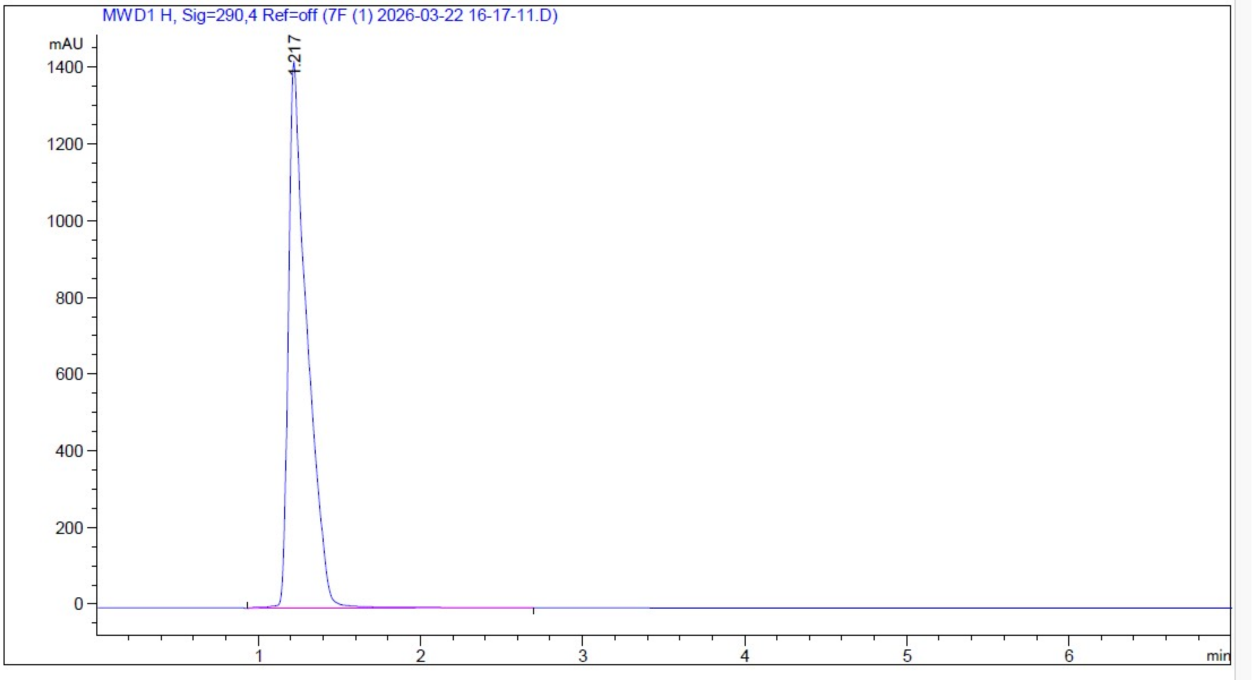

HPLC chart of compound 7f

Experimental procedures and instruments

2 Material and methods

2.1 Bioinformatics study

2.1.1 Identification of related targets for anti-epilepsy and Prediction of compound targets

Targets associated with anti-epilepsy were retrieved from GeneCards platform (https://www.genecards.org/). All genes retrieved from GeneCards were used as the input gene set. Initially, 2086 genes were collected. After merging and filtering, the final gene set contained 1853 genes. Some genes had low GeneCards scores, but their names were still included; no additional score-based filtering was applied. This final set was used for downstream analyses. The potential molecular targets of the active compounds (**6b, 6c, 6d**) and (**7a, 7e, 7f**) were predicted using Swiss Target (http://www.swisstargetprediction.ch/), Prediction with Homo sapiens selected as the target species. For each compound, the top 100 predicted targets ranked by probability score were retained for subsequent analysis. specifying homo sapiens as the target species. The intersection of anti-epilepsy -related targets and compound-associated targets was determined and visualized using VENNY 2.1.0 (https://bioinfogp.cnb.csic.es/tools/venny/), to identify common targets potentially involved in the antiepileptic activity of the compounds. These overlapping genes were considered the input gene set for subsequent enrichment analysis and downstream target prioritization.

2.1.2 Gene ontology (GO) functional annotation and Kyoto Encyclopedia of Genes and genomes (KEGG) pathway analysis

The GO functional annotation and KEGG pathway enrichment analyses were performed using the ShinyGO platform.(http://bioinformatics.sdstate.edu/go/) is a web-based tool designed for the analysis of gene expression data, focusing on GO terms, pathway analysis, and protein–protein interaction networks. To evaluate the predicted antiepileptic effects of the investigated compounds, enrichment analyses were performed using KEGG biological process enrichment analysis of predicted target genes associated with the investigated compounds (nGenes indicates the number of predicted target genes involved in each pathway), GO biological processes, and GO molecular functions. Pathways with a false discovery rate (FDR) < 0.05 were considered statistically significant

2.2 Experimental animals

Male Swiss albino mice weighing 23-27 g were used in this study. The animals were housed in standard polypropylene cages under controlled laboratory conditions: ambient temperature 22 ± 2 °C, relative humidity 50-60%, and a 12-hour light/dark cycle. Standard rodent pellet chow and water were provided ad libitum throughout the experiment. To minimize stress and allow physiological acclimatization, mice were allowed to adapt to the laboratory environment for one week prior to any experimental procedures. All animal experiments were conducted in accordance with institutional and national ethical guidelines for animal research and were approved by the Ethics Committee of the Faculty of Pharmacy, Egyptian Russian University (Approval Code: ERUFP-PC-25-001).

2.3 PTZ-induced seizure model

For the PTZ screen, a total of 80 mice were randomly divided into eight groups (10 mice per group). The groups were organized as follows: (1) a PTZ control group receiving 0.5% carboxymethyl cellulose (CMC) vehicle orally, 30 minutes prior to PTZ injection; (2) a reference group treated with sodium valproate (300 mg/kg, p.o., as a positive control); and (3-8) six test groups administered with compounds **6b, 6c, 6d, 7a, 7e,** and **7f** (20 mg/kg, p.o. for each compound). Thirty minutes after the pretreatment, all groups (except the normal vehicle control) were challenged with a convulsive dose of pentylenetetrazol (85 mg/kg, i.p.) to induce seizures. Following PTZ administration, mice were placed individually in transparent observation chambers, and an observer blinded to the treatment recorded seizure activity for each animal.

The anticonvulsant efficacy of each treatment was quantified by assessing: (a) the seizure protection rate, defined as the percentage of mice in the group that did not develop generalized tonic-clonic seizures; (b) the relative protection compared to the valproate reference group, reflecting the potency of each test compound in preventing seizures relative to the standard drug; and (c) the mortality rate within 24 hours post-PTZ, as an indicator of severe seizure outcomes.

2.4 Pilocarpine-induced seizure model

Mice were assigned to nine groups (n = 10 per group) for this phase: (1) a normal control group that received vehicle (no pilocarpine) and served as a baseline; (2) a pilocarpine control group that was given pilocarpine (300 mg/kg, i.p.) to induce status epilepticus; (3) a sodium

valproate group (300 mg/kg, p.o.) serving as a positive control; and (4-9) six experimental groups pretreated with the test compounds (each at 20 mg/kg, p.o.). All mice, except those in the normal control group, were injected with pilocarpine (300 mg/kg, i.p.) to induce seizures, 30 minutes after administration of either valproate or test compounds. To mitigate peripheral cholinergic effects of pilocarpine, all animals receiving pilocarpine were pretreated with the peripheral muscarinic antagonist hyoscine butylbromide (1 mg/kg, i.p.) 20 minutes before the pilocarpine injection.

Following pilocarpine administration, animals were observed for a 120-minute period for signs of seizures. Seizure activity was evaluated by recording: (a) the latency to seizure onset, defined as the time (in minutes) from pilocarpine injection to the first appearance of a Stage 3 seizure (forelimb clonus) on the Racine scale; (b) the seizure severity progression, monitored by scoring each animal's seizure activity at 30-minute intervals according to Racine's five-point scale (0 = no response, 1 = facial automatisms, 2 = head nodding, 3 = forelimb clonus, 4 = rearing and falling, 5 = generalized tonic-clonic seizure); and (c) the 24-hour survival rate for each group following status epilepticus, as a measure of protection against lethal seizures. At the end of the 2-hour observation period, animals that survived were humanely euthanized, and the brains were quickly removed to harvest hippocampal tissues from select groups for biochemical analysis. In this study, hippocampal samples were collected from the normal control group, the pilocarpine control group, valproic acid group, and the group treated with the most effective test compound (6d). These tissue samples were immediately frozen and later processed for assays to investigate the compound's effect on neurochemical markers of seizure-induced damage.

2.5 Evaluation of hippocampal biochemical changes

Hippocampal oxidative stress was evaluated by measuring the levels of malondialdehyde (MDA) and nitric oxide (NO) in tissue homogenates. MDA was quantified as an index of lipid peroxidation, whereas NO levels were determined indirectly by assessing its stable metabolite, nitrite, using the Griess reaction. Both MDA and nitrite concentrations were measured with commercially available colorimetric assay kits (Biodiagnostics Co., Giza, Egypt) according to the protocols provided by the manufacturer.

To investigate excitotoxic and inflammatory responses in the hippocampus, the concentrations of glutamate (an excitatory neurotransmitter) and pro-inflammatory cytokines

tumor necrosis factor-α (TNF-α) and interleukin-6 (IL-6) were determined in the harvested tissue. These biomarkers were quantified using specific enzyme-linked immunosorbent assay (ELISA) kits: glutamate was measured with a competitive ELISA kit (MyBioSource, San Diego, CA, USA; Cat# MBS756400), while TNF-α and IL-6 were measured with sandwich ELISA kits (TNF-α: CUSABIO, Houston, TX, USA; Cat# CSB-E11987r; IL-6: R&D Systems, Minneapolis, MN, USA; Cat# R6000B). Hippocampal tissue homogenates were prepared and centrifuged at 12,000 × g for 15 minutes at 4 ℃, and the resulting supernatants were used for the assays according to the kit manufacturers' instructions. Total protein content in each sample was determined by the biuret method to allow normalization of glutamate and cytokine levels per milligram of protein.

2.6 Toxicity and safety evaluation

The safety profile of the lead compound 6d was examined using a sub-chronic dosing regimen in mice. Male mice were administered 20 mg/kg, p.o. doses of 6d once daily for 14 consecutive days, while control animals received the vehicle alone (0.5% CMC, p.o.). Throughout the treatment period, all mice were closely monitored for any signs of toxicity, such as changes in general behavior, signs of discomfort or distress, and any mortality. At the end of the 14-day dosing period, all animals were anesthetized for blood collection via retro-orbital sinus puncture. Blood samples were allowed to clot and then centrifuged at 3000 rpm for 10 minutes to separate serum. The obtained serum was analyzed for key biochemical markers of organ function using standard diagnostic kits. Liver function was evaluated by measuring the activities of alanine aminotransferase (ALT) and aspartate aminotransferase (AST) with kinetic enzyme assay kits (Spectrum Diagnostics, Cairo, Egypt). Renal function was assessed by determining serum urea and creatinine levels (Spectrum Diagnostics kits), and potential cardiotoxicity was examined by measuring serum creatine kinase-MB (CK-MB) and troponin T levels by ELISA kits from LSBio (Cat# LS-F5746) and MyBioSource (Cat# MBS730382), respectively.

2.7 Animals Section

Animals were randomly allocated to experimental groups using simple randomization. Investigators responsible for seizure scoring and outcome assessment were blinded to treatment allocation during the experiment and data analysis. The approval code is: ERUFP-PC-24-001.

2.8 End of Statistical Analysis

For the carrageenan-induced paw edema model, celecoxib was used as reference anti-inflammatory drug, and comparisons were made against the carrageenan and celecoxib control groups. For the PTZ- and pilocarpine-induced seizure models, sodium valproate was used as the positive anticonvulsant control, and comparisons were made against the corresponding disease-control groups. For hippocampal biochemical analyses, normal control, pilocarpine control, valproic acid, and compound 6d groups were compared to assess seizure-associated oxidative stress, neuroinflammation, and excitotoxicity.

2.9 HPLC results

HPLC analysis was performed using an HPLC system (Agilent 1260 Infinity II, Agilent Technologies Inc., USA) equipped with an Agilent Eclipse XDB-C8 column (5 µm). All compounds were dissolved in methanol and injected at a volume of 20 µL. Detection was carried out at the λmax of each compound to ensure optimal sensitivity. The mobile phase composition was optimized individually for each compound to achieve adequate retention and peak separation, while a constant flow rate of 1.5 mL/min was maintained. Under these conditions, each compound produced a well-defined single peak, indicating suitable chromatographic performance and suggesting high purity of the compounds.

2.10 *In-silico* ADME study

The physicochemical descriptors, along with the predicted pharmacokinetic and drug-likeness profiles of the synthesized compounds, were assessed using the SwissADME and ProTox 3.0 web servers. In the early stages of drug development, it is imperative to evaluate these properties, as they play a critical role in determining the success of a candidate compound. Beyond potency, the absorption, distribution, metabolism, excretion, and toxicity (ADMET) characteristics, as well as overall drug-likeness, are essential considerations in optimizing lead compounds for further development. These parameters help predict the compound's behavior in biological systems, thereby facilitating the selection of candidates with favorable pharmacological profiles, in addition the target prediction survey was conducted with SwissTargetPrediction

2.11 Molecular Docking Studies

Molecular docking was employed to investigate the interactions between the synthesized ligands and the target protein, in comparison with a standard drug. Docking simulations were performed using the crystal structure of reduced human cytosolic branched-chain aminotransferase (VGSCs; PDB ID: 2COJ), obtained from the RCSB Protein Data Bank (https://www.rcsb.org/) in PDB format. Molecular docking was carried out using AutoDock 4.2 software. The resulting protein–ligand interaction complexes were visualized, and 2D interaction diagrams were generated using BIOVIA Discovery Studio Visualizer to analyze binding modes and key interacting residues

Reference

[1] E.M. Gedawy, A.E. Kassab, A.M. El Kerdawy, Design, synthesis and biological evaluation of novel pyrazole sulfonamide derivatives as dual COX-2/5-LOX inhibitors, European Journal of Medicinal Chemistry 189 (2020) 112066. https://doi.org/10.1016/j.ejmech.2020.112066.

[2] Y. Gao, S. Samanta, T. Cui, Y. Lam, Synthesis and in vitro evaluation of west nile virus protease inhibitors based on the 1,3,4,5-tetrasubstituted 1H-Pyrrol-2(5H)-one scaffold, ChemMedChem 8 (2013) 1554–1560. https://doi.org/10.1002/cmdc.201300244.
